# Immunoseq: the identification of functionally relevant variants through targeted capture and sequencing of active regulatory regions in human immune cells

**DOI:** 10.1186/s12920-016-0220-7

**Published:** 2016-09-13

**Authors:** Andréanne Morin, Tony Kwan, Bing Ge, Louis Letourneau, Maria Ban, Karolina Tandre, Maxime Caron, Johanna K. Sandling, Jonas Carlsson, Guillaume Bourque, Catherine Laprise, Alexandre Montpetit, Ann-Christine Syvanen, Lars Ronnblom, Stephen J. Sawcer, Mark G. Lathrop, Tomi Pastinen

**Affiliations:** 1 Department of Human Genetics, McGill University, Montréal, Quebec Canada; 2 McGill University and Genome Québec Innovation Centre, Montréal, Quebec Canada; 3 Department of Clinical Neurosciences, University of Cambridge, Cambridge, UK; 4 Department of Medical Sciences, Section of Rheumatology, Uppsala University, Uppsala, Sweden; 5 Department of Medical Sciences, Molecular Medicine and Science for Life Laboratory, Uppsala University, Uppsala, Sweden; 6 Département des sciences fondamentales, Université du Québec à Chicoutimi, Saguenay, Quebec Canada

**Keywords:** Rare variants, Immune disease, Gene expression, Next-generation sequencing, Capture

## Abstract

**Background:**

The observation that the genetic variants identified in genome-wide association studies (GWAS) frequently lie in non-coding regions of the genome that contain *cis-*regulatory elements suggests that altered gene expression underlies the development of many complex traits. In order to efficiently make a comprehensive assessment of the impact of non-coding genetic variation in immune related diseases we emulated the whole-exome sequencing paradigm and developed a custom capture panel for the known DNase I hypersensitive site (DHS) in immune cells – “Immunoseq”.

**Results:**

We performed Immunoseq in 30 healthy individuals where we had existing transcriptome data from T cells. We identified a large number of novel non-coding variants in these samples. Relying on allele specific expression measurements, we also showed that our selected capture regions are enriched for functional variants that have an impact on differential allelic gene expression. The results from a replication set with 180 samples confirmed our observations.

**Conclusions:**

We show that Immunoseq is a powerful approach to detect novel rare variants in regulatory regions. We also demonstrate that these novel variants have a potential functional role in immune cells.

**Electronic supplementary material:**

The online version of this article (doi:10.1186/s12920-016-0220-7) contains supplementary material, which is available to authorized users.

## Background

Genome-wide association studies (GWAS) have identified thousands of associated single nucleotide polymorphisms (SNPs) in hundreds of complex diseases [1] and have thereby provided unprecedented insights into the genetic architecture underlying these conditions [2]. However, because GWAS are inherently dependent upon there being meaningful linkage disequilibrium (LD) between relevant variation and the few hundred thousand common variants that are actually genotyped this method has limited ability to accurately assess the role of rare variants [3] and effectively only screens common variation [4]. This limitation has been suggested to contribute to the notable gap between observed heritability and that explained by the currently identified common variants - the so-called missing heritability [5]. Direct assessment of all variation through the next-generation sequencing of the whole genome would provide a comprehensive assessment that would necessarily avoid any dependency on LD but unfortunately remains prohibitively expensive. On the other hand the targeted capture of genomic regions with high prior probability of containing relevant variation allows next-generation sequencing efforts to be focused and therefore substantially more affordable. This logic underlies whole exome sequencing which allows comprehensive assessment of coding variation and has enabled the identification of rare coding variants exerting large effects in a number of complex diseases [6–8]. It is notable that the majority of the associated variants identified through immune disease GWAS are located in non-coding regions of the genome that are enriched for regulatory elements that are active in immune cell types [9–11], suggesting that a resequencing effort focused in these regulatory regions would provide a highly efficient means to identify both common and rare variation of relevance in such diseases.

Using deoxyribonuclease I (DNase I) based sequencing (DNase-seq) international collaborative efforts such as the ENCODE [12] and NIH Roadmap Epigenomics [13] projects have established comprehensive maps of DNase I hypersensitive sites (DHSs) in multiple cell types. Sites which are markedly enriched for *cis*-regulatory elements active in those cell types such as enhancers and promoters [14, 15], show very high concordance with chromatin immunoprecipitation sequencing of histone marks for active enhancers or promoters [16, 17] and are enriched for SNPs (eSNPs) that influence the expression of local genes that show variable expression (expression quantitative trait loci, eQTLs) [16, 18, 19]. It has been noted that the enrichment of eSNPs is most pronounced in those functional elements that are located closest to their respective eQTL [17] and that there might be an inverse relationship between the effect size of *cis*-eQTLs and the minor allele frequency (MAF) of the relevant eSNP; suggesting that rare variants might have a higher impact on gene expression than common variants [20–23].

Based on the overwhelming evidence from GWAS that common variants associated with immune disease likely influence disease risk by perturbing the regulation of gene expression together with emerging evidence indicating the existence of rare “high-impact” non-coding variation, we designed a custom capture panel, relying on contemporary regulatory element maps, to enable the targeted re-sequencing of immune regulatory regions - “Immunoseq”. Immunoseq is designed to allow efficient re-sequencing of regulatory regions of relevance in immune cells (coding and non-coding) and thus enable a comprehensive assessment of all potentially relevant variation in these regions, both common and rare. The panel includes SNPs previously associated with immune traits as well as established immune cell eSNPs. Using Immunoseq in parallel with transcriptome sequencing (RNA-seq), we show that, after accounting for effects attributable to associated common variants, there are significant effects attributable to rare variants, and that these explain up to 14 % of residual variation. Our results confirm that targeted capture and re-sequencing of regulatory regions active in relevant cell types provides an efficient means to identify rare variants of relevance in immune disease.

## Methods

### Design of the Immunoseq custom capture panel

We selected regulatory regions of immune cells using genome-wide DHS data from the ENCODE [12] and NIH Roadmap Epigenomics [24] projects. Data from 12 different immune cell types were utilized: CD3+, CD3+ cord blood, CD4+, CD8+, CD14+, CD19+, CD20+, CD34+, CD56+, Th1, Th2, Th17 (Additional file 1: Table S1). The entire genome was divided in 100 bp bins and the DHS signals were normalized by calculating the number of reads per bin divided by the total number of reads. In each sample, the signals were ranked and the top 300,000 bins (representing the top 1 % of the genome) identified, within each cell type bins were retained if they were identified in at least 50 % of the available samples. For those cell types where only two samples were available, the selected bins were required to be present in both samples; in those cell types where only one sample was available (Th2, Th17 and CD20) all 300,000 bins were retained. The 100 bp bins were then grouped into blocks of 50,000 bins each (i.e. 0 to 50,000 top bins, 50,000 to 100,000 top bins etc.) and when the overlap between sample blocks (from the same cell type) dropped below 50 %, the blocks were eliminated. Additional file 1: Table S1 shows the number of bins used and the number of samples available for each cell type. All selected regions were combined and bins were removed when at least 50 % of a bin overlapped with an exome capture region (SeqCapEZ Exome V3 Capture, Roche, 64.1 Mb). Non-coding regions targeted by our design cover a total of 67.3 Mb. The Immunoseq custom capture was complemented by exome (SeqCapEZ Exome V3 Capture, Roche, 64.1 Mb) and Human Leukocyte Antigen (HLA) regions (SeqCap EZ design, Human MHC design from Roche, 4.97 Mb) totaling 138 Mb for the panel.

### Enrichment of GWAS hits in DHSs selected for the Immunoseq custom capture panel design

GWAS hits were obtained from the National Human Genome Research Institute (NHGRI) (https://www.ebi.ac.uk/gwas/, January 29^th^ 2015). We selected SNPs from different disease categories: Immune and chronic inflammatory diseases (724 SNPs), associated to more than one immune or chronic inflammatory diseases (49 SNPs), Neuropsychiatric disease (65 SNPs) and Cancer (393 SNPs), including SNPs in LD using HaploReg V2 (*r*^2^ > 0.9) [25]. Functional variants were selected from Monocyte and B-cell *cis-*eQTLs identified in the paper by Fairfax and colleagues [18]. Associated eQTLs with empirical p < 0.001 after 1000 permutations, for each top hit per transcript were retained for each cell type. ImmunoChip hits (224 SNPs) from five immune and chronic inflammatory disease studies [26–30] were used. The analysis of overlap between Immunoseq regions and SNPs was determined using bedtools (v2.17.0).

We compared the enrichment of GWAS or ImmunoChip hits and functional variants in DHS regions included in the Immunoseq to other regions: 1) DHS from other cell types (Additional file 1: Table S2) selected in the same way as for the immune cells in the Immunoseq design, 2) Same as in 1) but keeping only regions that do not overlap immune cell DHS regions selected for Immunoseq (compared to Immunoseq DHS regions not overlapping with the other cell types’ DHS regions), 3) an equal number of bins as in the Immunoseq DHSs selected randomly from the whole genome in 1000 iterations and 4) an equal number of bins as in the Immunoseq DHSs selected randomly from the non-coding genome in 1000 iterations. For the randomly selected regions, the whole genome was split into 100 bp bins and 67,300 of them were selected, 1000 times. Fisher’s exact test was performed to evaluate the significance of the enrichment.

### Design of the second version of Immunoseq

Using coverage statistics from the first version of the Immunoseq panel, we flagged poorly covered regions (<0.1X across all samples) or unusually high coverage regions (>120x across all samples), as well as ENCODE Blacklist regions for removal, and used the remaining regions to begin designing a 2^nd^ version of our Immunoseq panel. Additional regions totalling 7.243 Mb based on Digital Genomic Footprinting (DGF) data from ENCODE for CD4+, CD8+, CD19+ and CD56+ were added for this new panel.

### Capture and sequencing

Thirty samples from the Swedish Uppsala Bioresource cohort were used as the discovery sample set in this study. The regional ethical review board in Uppsala, Sweden approved the study and all participants gave their informed consent. The Cambridge Multiple Sclerosis (MS) sample set was used as a replication set in this study. Eighty-six affected and 94 healthy controls were included for a total of 180 samples. DNA was prepared from Peripheral Blood Mononuclear Cells using standard methods. DNA quantification was performed using PicoGreen.

Whole-genome library preparation was performed using 500–1000 ng of genomic DNA. Covaris focused-ultrasonicator E210 was used for shearing DNA into 150–1500 bp fragments. LabChip EZ reader was used for fragment size evaluation and size selection was performed when needed. Libraries were prepared using the KAPA High Throughput (HTP) Library Preparation Kit (KAPA Biosystems). The end repair to produce blunt-ended double stranded DNA, adenylation of the 3′-ends, adapter ligation and amplification were performed following the recommendations from the kit manufacturer and cleaned using AMPure XP beads. The libraries were analyzed on LabChip and quantified using PicoGreen. Samples were then pooled (2X, 5X or 6X) using a total of 1 μg of library, followed by Roche NimbleGen SeqCap EZ Library instructions for the hybridization of the baits and the capture steps. The final amplification was done using KAPA HTP. Concentration, size distribution, and quality of the amplified capture were assessed using LabChip. Captured products were sequenced on the Illumina HiSeq2500 or HiSeq2000 with 100 bp paired-end reads. The discovery sample set was captured with the first version of the panel, and the replication set was captured with the second version of the panel. For the second panel, the library preparation and capture steps were automated and performed using the Biomek FX (Beckman Coulter).

### Read mapping and variant calling

Reads were aligned to Genome Reference Consortium Human genome build 37 (GRCh37) using bwa 0.7.6a. and variants were called using HaplotypeCaller v3.2 (GATK).

### Variants quality control/SNVs validation

Quality cut-off was set at read depth ≥10, genotyping quality (gq) ≥70, and mapping quality (MQ) ≥50. These cut-offs were selected based on the comparison of the sequencing and genotyping data (Human Omni2.5 BeadChip in the 30 sample cohort or Human Omni5 BeadChip in the 180 sample set), available for all samples, where both had concordance of over 95 % (Additional file 1: Figure S1). Indels were not included in our analysis.

To test the variant capture efficiency of Immunoseq, we applied our panel to a Yoruban sample (NA18502) that has been sequenced at high depth by Complete Genomics [31]. We compared the accuracy of the heterozygous variants identified by Complete Genomics that overlapped with the panel regions with the variants identified using our custom capture panel (Additional file 1: Figure S2). DNA sequencing data from the NA18502 sample was downloaded from the public genome data repository (ftp2.completegenomics.com, assembly software version 1.10).

### Annotation of variants

The GERP++ score was used as a metric for conservation to identify selectively constrained variants (http://mendel.stanford.edu/SidowLab/downloads/gerp/) [32]. We also used the CADD tool to score the deleteriousness of the identified variants (http://cadd.gs.washington.edu/) [33]. Coding variants were annotated using snpEff [34]. Common variants are defined as having MAF > =1 % and rare variants are defined as having MAF < 1 % based on the allele frequencies from the 1000 Genomes Project [35]. Novel variants were defined as variants not observed in the 1000 Genomes Project or dbSNP141.

### Shared vs cell-type specific DHSs

The DHS sets selected for each cell type were intersected to determine which bins are observed in all selected cell types or in a subset of the cells. Enrichment was measured by comparing the number of rare and/or novel variants to the number of common variants falling in each category of DHSs and the total observed in DHSs.

### Identifications of variants that disrupt or create motifs

Each identified variant was tested for the impact of the reference and the alternate allele on transcription factor motifs ± 15 nucleotides from the variant position. Matrices for TRANSFAC (version 2009.4) were used with the Finding Individual Motif Occurrence (FIMO) scanning software, version 4.10.1, using a p < 1.42e-7 threshold (Bonferroni correction: 0.05/351,088 SNPs =1.42e-7). Only motifs directly overlapping a variant were kept. A motif was considered as created if it had a significant matrix affinity score only with the alternate allele, whereas it was considered disrupted if it had a significant matrix affinity score only with the reference allele.

### RNA-sequencing and allele-specific expression mapping

Purified T cells were isolated from the discovery set samples (eight CD3+ and 20 CD4+). RNA was isolated with miRNeasy Mini Kit (Qiagen) and 500 ng of RNA was used to prepare libraries using Illumina TruSeq Stranded Total RNA Sample preparation kit following the manufacturer’s instructions. Quality control was performed using Agilent Bioanalyzer and samples were sequenced on Illumina HiSeq2000 with 100 bp paired-end reads. Raw reads were trimmed (quality: phred33 ≥ 30 and length *n* ≥ 32), adapters were removed (using Trimmomatic V.0.32 [36]) and reads were aligned to the hg19 human reference (Tophat v.2.0.10 [37] and bowtie v.2.1.0 [38]) for 81.9 % of the reads aligned. For the replication set, purified T-cell (CD4+ and CD8+) subpopulations were isolated from 180 subjects (86 multiple sclerosis patients and 94 healthy controls) for 73 % of the reads aligned. For details see Lemire et al. [39].

Allele counts were measured using the SNPs from Illumina Human Omni2.5 BeadChip (30 samples cohort) or Human Omni5 BeadChip (180 samples cohort) and imputation (1000 Genomes Project, using the IMPUTE2 software). Haplotype phasing was performed using the SHAPEIT V2 software and allele specific expression was calculated using reads from whole genes as previously described [19]. We used the Allele-specific expression (ASE) association data calculated with the replication cohort for the first cohort because of the lack of power due to the small samples number. Since CD3+ cells were not assessed in the replication cohort, we use the combination of CD4+ and CD8+ data to get association *p*-values for this cell type. Transcripts with association *p*-value <1e-5 were kept, and isoforms were removed based on normalized read counts for each gene (keeping the best covered isoform). A total of 3859 transcripts for CD3+ cells and 3428 transcripts for CD4+ cells in the 30 samples discovery set, and 5536 transcripts for CD4+ and 5594 transcripts for CD8+ cells in the replication set were included in the analysis.

### Enrichment of rare variants in vicinity of allelically imbalanced (AI) genes

The fold difference between the expressed alleles was calculated as counts for the most abundant allele divided by counts for the less abundant allele. Thus a fold difference of one corresponds to alleles that are expressed equally. Genes with fold difference between 2 and 9 were considered as having allelic imbalance (AI). Genes with > 9-fold were considered to be enriched for imprinted loci or artefacts and were thus removed from the analyses.

We performed enrichment analysis for variants in DHS +/−20 kb from each gene. We calculated the enrichment of rare variants in highly AI genes (ASE effect size between 2 and 9, 1 meaning both alleles are expressed equally) by dividing the proportion of AI genes with rare variants in correlated DHSs by the proportion of all tested genes with rare variants in correlated DHSs.

### DNAse –sensitive regions correlated to transcript promoters

NIH ENCODE Roadmap DHS datasets (*n* = 317) were retrieved and binned into 100 bp segments as described above. Using transcripts from GENCODE v15, we extracted all promoter regions (defined as transcription start site (TSS) +/−500 bp). Across all of the DHS datasets, we correlated the normalized bin scores for these promoter region bins with all DHS bins +/− 1 Mb.

### Hi-C region linked with promoter regions

Hi-C data from GM12787 lymphoblastoid cell line were obtained from Rao et al. [40] (Gene Expression Omnibus accession number: GSE63525). We extracted all regions that overlapped promoter regions (1500 bp from TSS) of gene where expression data was available, as well as the linked regions.

## Results

### Design of the Immunoseq custom capture panel

In order to select the most relevant non-coding regions to target, we used DNase I mapping data available from the ENCODE and Roadmap epigenomics projects from 12 different cell types (CD3+, CD3+ cord blood, CD4+, CD8+, CD14+, CD19+, CD20+, CD34+, CD56+, Th1, Th2 and Th17, Additional file 1: Table S1) [12, 13]. The whole genome was divided into 100 base pair bins, which were ranked according to the DHS signal for all samples available for every immune cell type (Methods). The top 300,000 signal intensity bins for every cell sample from the ENCODE and Roadmap epigenomics project were used for the design of the Immunoseq capture panel. The bins that were kept were required to be consistent in most (>50 %) biological replicates used for each cell type. Additional file 1: Table S1 shows the number of DHS signal intensity bins used and the number of samples available for every cell type. We combined these putative regulatory regions (67.3 Mb) with the coding regions from exome capture and the HLA region. However, given the unique and complex role of HLA in immune disease risk along with the extreme sequence diversity of human Major Histocompatibility Complex, we exclude its analysis in the following discussion. Altogether, Immunoseq covers a total 138 Mb of the genome.

### The Immunoseq regions are enriched in pertinent GWAS hits and eQTLs

We estimated the sensitivity of this panel by determining the extent to which it captured known autoimmune and chronic inflammatory diseases associated SNPs listed in the National Human Genome Research Institute (NHGRI) GWAS catalogue (*p* < 5 × 10^−8^) [1]; or SNPs in high linkage disequilibrium (*r*^2^ > 0.9) with these [25] (Additional file 1: Table S1-S2). We repeated this process using cancer and neuropsychiatric diseases associated SNPs listed in the GWAS catalogue (assuming that immune cells play a less significant role in these conditions, although it some case, it can play one) and using *cis-*eQTL data for monocytes (CD14+) and B-cells (CD19+) from Fairfax et al. [18].

**Fig. 1:**
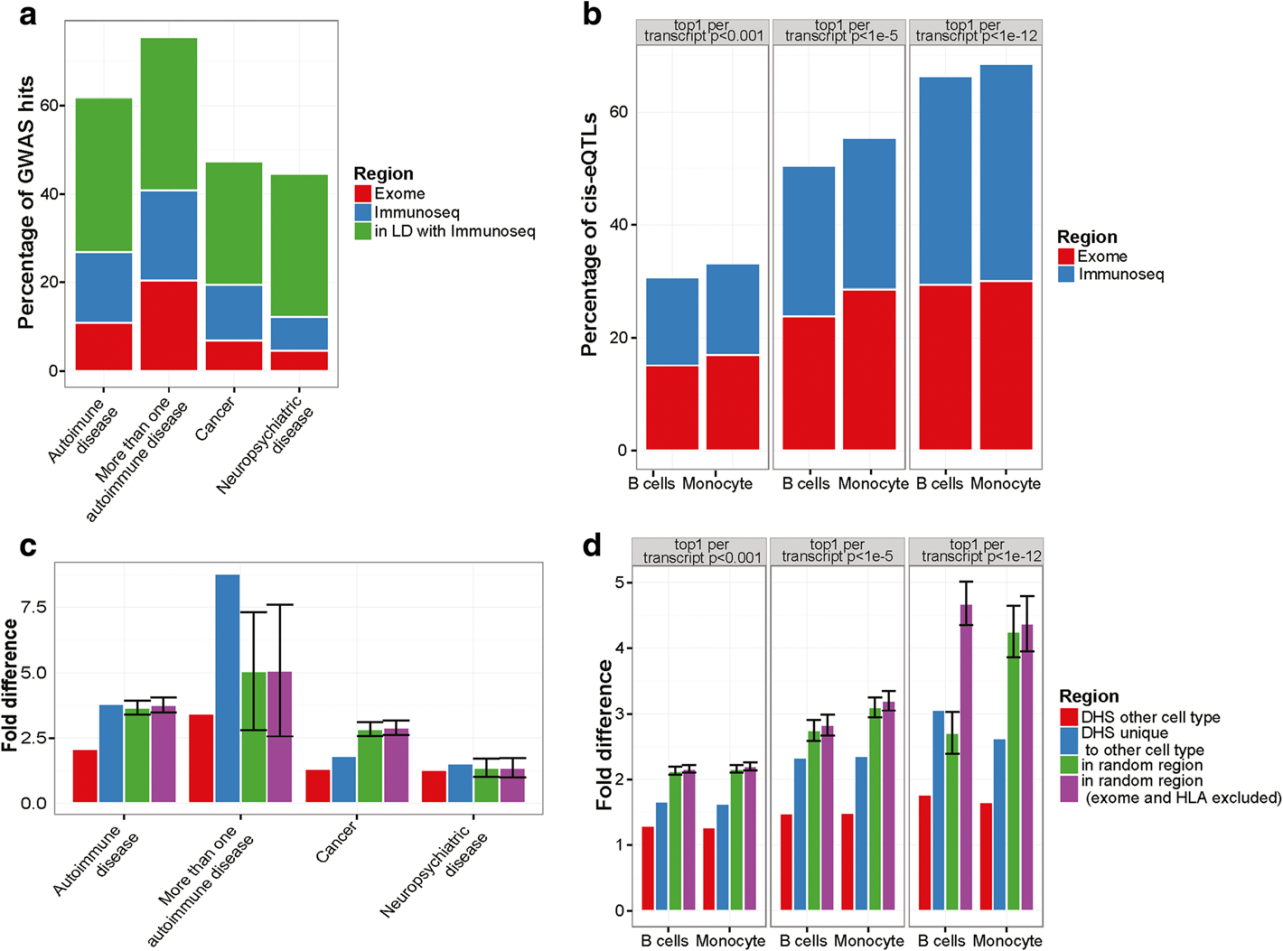
Benchmarking the ImmunoSeq capture panel by known disease associated sites and regulatory variants. **a** Autosomal GWAS hits associated to more than one autoimmune or chronic inflammatory disease, for neuropsychiatric diseases and for cancer included in the Immunoseq. custom capture panel. (Cut-off of 1 × 10^−8^ was used to select GWAS hits to analyze, SNPs in LD selected based on *r* ^2^ > 0.9, HLA (human leucocyte antigen) hits and region as well as chromosome X SNPs were excluded from the analyses). SNP in LD = GWAS hits that have a SNP in LD in the Immunoseq. custom capture panel. **b** cis-eQTLs from monocytes (CD14+) and B Cells (CD19+) (considered has haplotype block, *r* ^2^ > 0.9) included in the Immunoseq. panel. Cut-off of *p* < 1e-3 or *p* < 1e-5, and *p* < 1e-12 after 1000 permutations (1000 = number of SNPs tested per probe) and top 1 eQTLs per transcript were kept for analysis (HLA hits and region as well as chromosome X hits were excluded in the analyses). **c** Enrichment of GWAS hits (same as in A) and proximal SNPs (LD *r* ^2^ > 0.9) that fall in DHSs selected for immune cell types compared to DHSs selected from other tissues (either all or non-overlapping ones) and regions randomly selected (1000 times) from the whole genome (either the full genome or only non-coding regions excluding HLA). Significance was calculated using Fisher’s exact test. Enrichment is significant (*p* < 0.001) for all GWAS hits except for Neuropsychiatric hits. **d** Enrichment of eQTLs (same as in B) and proximal SNPs (LD *r* ^2^ > 0.9) positioned at DHSs selected for immune cell types compared to DHSs selected from other tissues (either all or non-overlapping ones) and regions randomly selected (1000 times) from the whole genome (either entire genome or only the non-coding part excluding the HLA region). All enrichments shown are significant (*p* < 0.001). All *p*-values were calculated using Fisher’s exact test

This panel includes SNPs in high LD (*r*^2^ > 0.9) with 62 % (448 SNPs) of the autoimmune disease associated variants listed in the GWAS catalogue (Fig. 1a), 63 % (140 SNPs) of the associated variants identified in key ImmunoChip studies [26–30] (Additional file 1: Figure S3A) and more than 68 % (378 SNPs) of the eSNPs identified by Fairfax et al. (Fig. 1b) [18]. These observations indicate the potential of our design to identify variants associated to autoimmune disease as well as other variants with potential functional impact on immune cell function. In contrast, alternate panels based on DHSs from randomly selected tissues, or random genomic regions show significantly poorer performance (Fig. 1c-d, Additional file 1: Figure S3B).

### Functional potential of rare and novel variants identified using Immunoseq

Performing Immunoseq on DNA from 30 healthy blood donors (Table 1) at a mean sequencing coverage of 52x, we found that on average 88 % of the reads were located on or near target, >98 % of the target regions were covered (only 1.90 % of the bases were missing) and 95 % of the target regions were covered by at least two reads.

**Table 1:** Sequencing statistics of the samples sequenced with Immunoseq

	Mean target coverage	Bases on target (%)^a^	Target region without coverage (%)^b^	Target bases with > =10x coverage (%)^c^	Level of multiplexing	Sequencing platform
Sweden Uppsala Bioresource samples (*n* = 30)	52X	88	1.9	83	2X (3 samples)	HiSeq2500 (2X samples)
					5X (27 samples)	HiSeq2000 (5X samples)

Alignment to the human hg19 reference genome, and variant calling (HaplotypeCaller) to identify all SNPs were performed. Shows average values across samples

^a^On and near bait bases/good quality bases aligned (according to Picards metrics). ^b^The percentage of target region that did not reach 2x coverage over any base.^c^The percentage of all target bases achieving 10X or higher coverage. We considered a variant to be true at > =10 depth

Taking advantage of the high sequencing depth, we were able to identify rare and novel variants at high confidence. We defined rare variants as those having a MAF <1 % in 1000 Genomes Project data (Phase3) and novel variants that have not previously been identified by either 1000 Genomes Project or dbSNP141. A total of 351,088 variants were identified, of which 275,042 were common, 50,004 were rare and 26,042 were novel (Table 2, Additional file 1: Table S3 and S4, Additional file 2: Table S5).

**Table 2:** General characteristics of the common, rare and novel single nucleotide variations (SNVs)

Total number (average per sample)		All	Common	Rare	Novel^a^
All (Immunoseq)		351,088 (90,594)	275,042 (83,839)	50,004 (5318)	26,042 (1437)
Coding^b^	All	60,946 (15,169)	45,545 (1818)	12,452 (1166)	2949 (185)
	Non-synonymous^c^	30,967 (7174)	21,807 (6403)	7405 (669)	1755 (102)
	Synonymous^c^	29,214 (7770)	23,434 (7305)	4785 (395)	995 (71)
	Stop-gained^c^	395 (71)	202 (56)	135 (13)	58 (2)
Exome^d^		120,245 (30,682)	91,818 (27,916)	21,497 (2280)	6930 (486)
Non-coding^e^		290,142 (75,424)	229,497 (70,020)	37,552 (4152)	23,093 (1251)
All DHS^f^		195,182 (51,559)	154,154 (48,056)	24,571 (2677)	16,457 (826)

Total number of variants and the average number of variants per sample that were included in the Immunoseq design

^a^Novel variants are defined as not identified in the 1000 Genomes Project nor included in dbSNP141. ^b^Coding variants are those located in the exons of the RefSeq coding sequence. ^c^Synonymous, non-synonymous and stop-gained variants were annotated using SNPeff and the hg19 version of the genome. ^d^The Exome is based on the Roche SeqCap EZ exome v3.0. ^e^ Non-coding variants are those not in the RefSeq coding sequence. ^f^ The All DHSs category combines all DHSs from the selected 12 cell types and could partly overlap with the Exome. Cut-offs used for the quality control of the variants are read depth ≥ 10, genotyping quality (gq) ≥ 70, mapping quality (MQ) ≥50, and proportion of the reference allele between 10 and 90 %

**Fig. 2:**
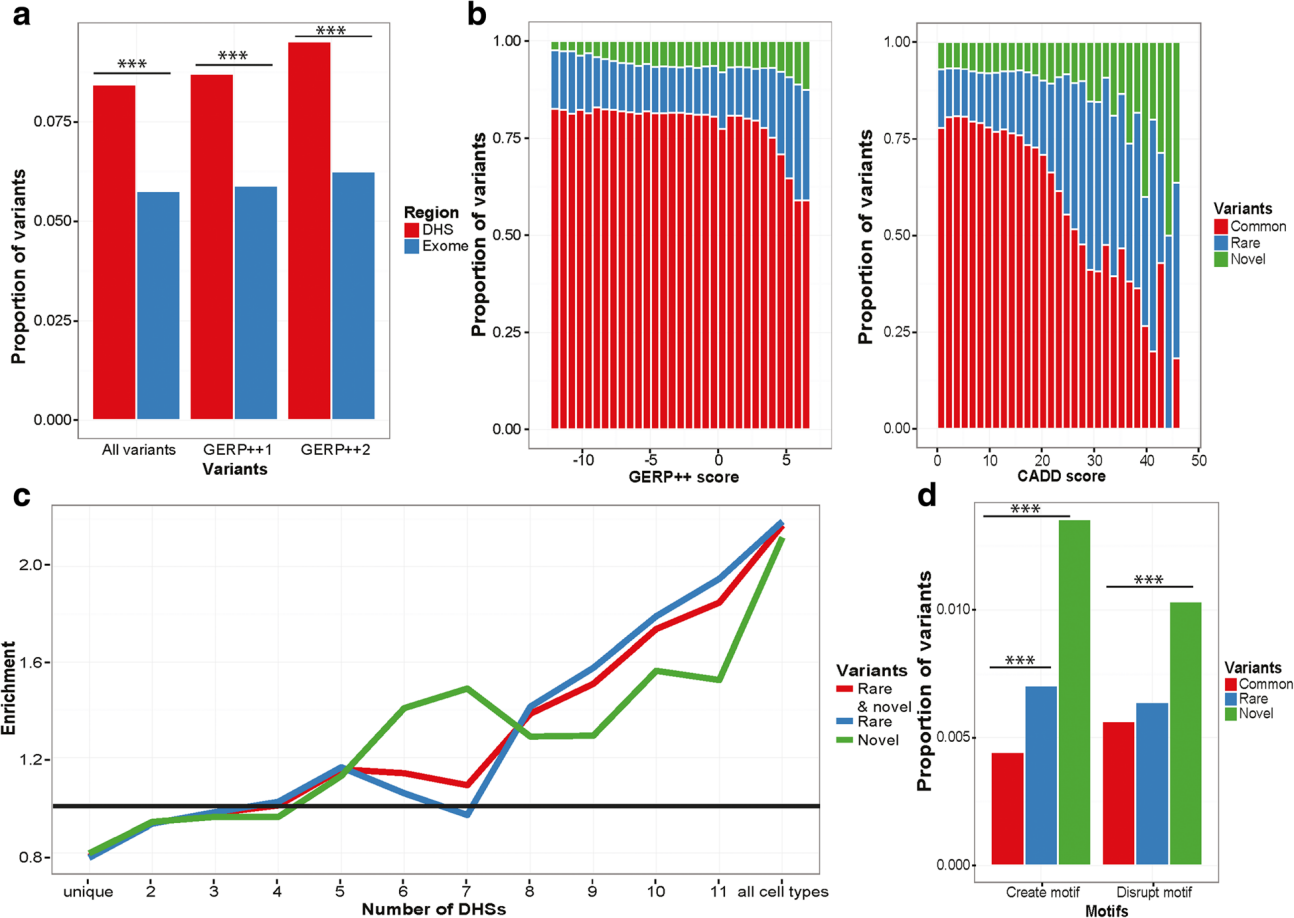
Discovery and functional potential of rare and novel variants using Immunoseq. **a** Proportion of novel variants (all, Genomic Evolutionary Rate Profiling (GERP++) > =1 and GERP++ > =2) identified in DHS (*red*) compared to the exome (*blue*). **b** Distribution of proportion of common (*red*), rare (*blue*) and novel (*green*) variants according to GERP++ score and Combined annotation dependent depletion (CADD) score. **c** Fold enrichment of rare (*blue*), novel (*green*) or rare and novel combined (*red*) variants compared to common variants found at shared or cell-type specific DHSs. Linear regression slope: rare =0.119 *p*-value = 1.35e-05, novel = 0.093 *p*-value = 5.81e-05, rare and novel = 0.113 *p*-value = 2.41e-06. **d** Proportion of common (*red*), rare (*blue*) and novel (*green*) variants localized at a DHS that either disrupt or create a transcription-factor binding motif. *P*-values are calculated using Fisher’s exact test (***p < 0.001)

Comparing non-coding with coding variants we found a significantly higher proportion were novel (*p*-value = 2.87e-175) and selectively constrained variants based on Genomic Evolutionary Rate Profiling (GERP++ ≥1 *p*-value = 3.57e-60 and GERP++ ≥ 2 *p*-value = 3.06e-47) (Fig. 2a). Using GERP++ [32] and Combined Annotation Dependant Depletion (CADD) scores [33], we also observed that the proportion of selectively constrained variants was greater amongst the novel and rare variants than amongst the common variants (Fig. 2b).

We next partitioned the variants called according to whether the DHS used in the design was shared among cell types or unique to one cell type. It has been previously shown that cell-type specific DHSs mostly overlap gene bodies and intergenic regions, whereas DHSs that are shared between cell types overlap with more active regions and promoters [41]. We observed a higher proportion of novel and rare variants compared to common variants in DHSs that are shared between cell types, compared to the ones that are unique for a single cell type (Fig. 2c). A clear increase in enrichment is observed when variants present at cell type unique DHSs and variants that are in DHSs shared between two to 12 cell types are compared, with linear regression *p*-values of 1.35e-05, 2.41e-06 and 5.81e-05 for rare, novel and combined rare and novel variants, respectively. These findings indicate that rare and novel variants are enriched in more active genomic regions compared to common variants.

To further investigate the potential functional impact of the rare and novel variants in DHSs, we explored the proportion of variants that disrupt or create transcription-factor motifs, compared to common variants and GWAS hits (Methods). In comparison to common variants a significantly higher proportion of novel variants create (*p*-value = 1.56e-38) or disrupt (*p*-value = 2.43e-11) transcription factor motifs (Fig. 2d). Rare variants show a slightly lower, but still significant enrichment for created motifs than do common variants (Fig. 2d, *p*-values for disrupted motifs = 0.16 and created motifs = 2.77e-07).

### The functional impact on gene expression by variants identified using Immunoseq

Given that rare non-coding variants in regulatory genomic regions can exert large *cis*-eQTLs effects and demonstrate extreme allele specific expression (ASE) bias [22, 23] we assessed the extent to which the rare and novel variants identified using Immunoseq influenced gene expression in a second independent set of samples; T cells (both CD4+ and CD8+) from 180 individuals used in a parallel effort to map common SNPs resulting in ASE (Ban, Ge et al. manuscript in preparation), almost 400 RNA-seq datasets in total.

We also generated deep RNA-seq data from fractionated T cells (CD3+ or CD4+) obtained from the 30 individuals used initially. These data generated equivalent results, which are shown in the Supplementary materials (Additional file 1: Figures S4-S10).

**Fig. 3:**
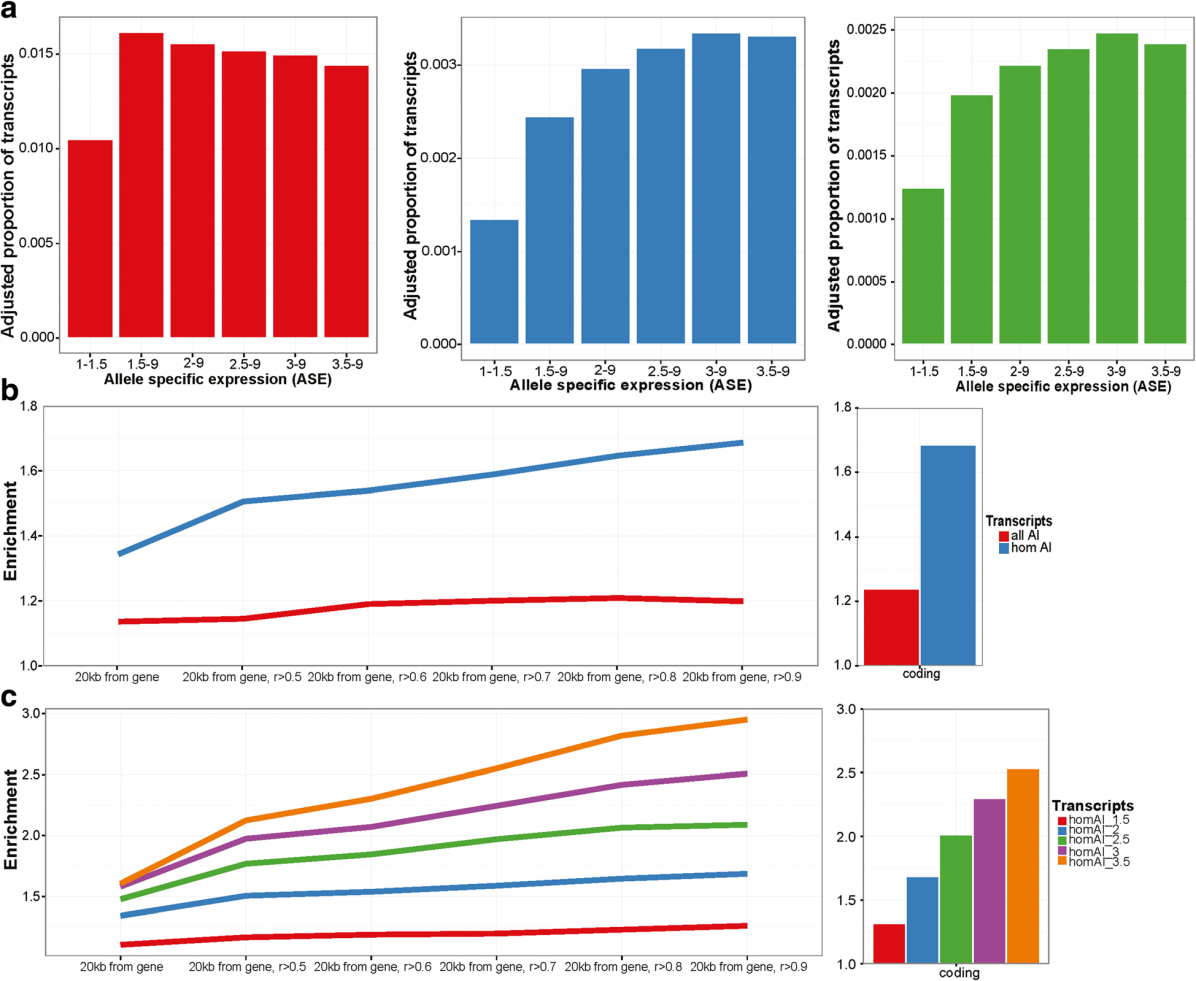
The impact of rare and novel noncoding variants on gene expression. **a** Using the replication set, we looked at the adjusted proportion of transcripts with common (*red*), rare (*blue*) or novel (*green*) noncoding variants in the vicinity (+/−20 kb) of a gene based on different allelic imbalance: 1.5 to 9, 2 to 9, 2.5 to 9, 3 to 9 and 3.5 to 9 fold difference. Adjustment was based on average number of SNPs used to calculate ASE at each ASE levels. **b** Enrichment of proportion of transcripts showing allelic imbalance (AI) with rare or novel variants in the vicinity of the gene compared to AI transcripts with common variants in vicinity of a gene. We looked at coding (histogram) vs noncoding variants as well as noncoding variants in DHS regions correlated with the promoters (Pearson correlation *r* > 0.5 to 0.9). In red are all transcripts where allelic imbalance was measured (allAI) and in *blue* are the transcripts for which the top associated SNP is homozygous in the sample (homAI). Linear regression slope for allAI = 0.015 (*p*-value = 0.0196) and homAI = 0.063 (*p*-value = 0.0024). Allelic imbalance genes are considered as > =2 fold between the alleles and equally expressed genes are < =1.5 fold. **c** Fold difference between proportions of AI transcripts with rare or novel variants in the vicinity compared to AI transcripts with common variants in the vicinity. Only including transcripts for which the top associated SNP is homozygous (homAI). We looked at coding (histogram) vs noncoding variants around the genes (+/−20 kb from gene) and in DHS regions correlated with the promoters (Pearson correlation *r* > 0.5 to 0.9). We compare different levels of allelically imbalanced transcripts from 1.5 fold to 3.5. all AI: AI transcripts comparing all transcripts for which ASE was measured and homAI: transcripts for which the top associated SNP that drives the association across samples is homozygous

For each gene we counted and characterised (coding/non-coding and novel/rare/common) the variants lying in the immediate vicinity (gene +/−20 kb) and determined the allelic imbalance (AI) in expression observed in each transcript. After adjusting for the average number of SNPs used to calculate AI for each transcript, we observed a higher proportion of transcripts with non-coding variants in their vicinity for transcripts where the higher AI level is independent of a common, rare or novel regulatory variant (Additional file 1: Figures S11-S12). A distinct increase in the proportion of variants was observed by comparing equally expressed transcripts with a <1.5-fold difference in their allelic expression with transcripts displaying AI with an ≥1.5, ≥2, ≥2.5, ≥3 and ≥3.5 fold difference in allelic expression (Fig. 3a). The increase in AI is more pronounced for transcripts flanked by rare or novel variants than by common variants. In order to control for the influence of common variants we repeated this analysis focusing on just those genes which are known to undergo ASE (Ban, Ge, et al. manuscript in preparation) and for which we had already mapped the common SNP contribution to *cis-*regulation by ASE-mapping [42]. This approach allowed us to include just those individuals that are homozygous for the relevant common eSNP and thereby exclude the influence of these common variants (Additional file 1: Figure S13). The same trend was observed for such transcripts when analysis was based exclusively on data from individuals homozygous for the local established common variant eSNP (Additional file 1: Figure S14).

This observation was then confirmed when rare and novel variants are considered together and this situation is even more pronounced when focusing on individuals homozygous for the relevant eSNP (Fig. 3b). Rare and novel variants located in DHSs that are correlated to the transcript promoters are highly enriched transcripts with substantial AI (> = 2 fold) compared to common variants, especially in transcripts with homozygous common eSNP (Fig. 3b). The stronger the correlation between a promoter and a DHS is, the more it is enriched in rare and novel variants (*p*-value = 0.0196), and is even stronger when looking at transcripts with homozygous common eSNP (*p*-value = 0.0024, Fig. 3b). We also observed that the transcripts displaying higher AI show more enrichment for rare and novel variants in its vicinity, compared to common variants (Fig. 3c). This was also observed when looking at rare and novel variants located in regions linked to the gene promoter by Hi-C (Additional file 1: Figure S15). The same increasing trend for DHSs correlated with promoters is observed for transcripts with different levels of AI (Fig. 3c). However, the observed trend is not as strong in all transcripts (Additional file 1: Figure S16). Coding rare and novel variants especially in transcripts with homozygous eSNP also appear to have an impact on gene expression, as they are as enriched in coding regions compared to common variants (Fig. 3b). The effect is almost as strong as the one observed for non-coding variants located at DHSs highly correlated with the promoter (Pearson’s *r*^2^ > 0.9) (Fig. 3b). Also, a similar trend of significant increased AI is observed for coding variants in transcripts with homozygous eSNP (Fig. 3c, linear regression slope = 0.227, *p*-value = 0.018).

**Fig. 4:**
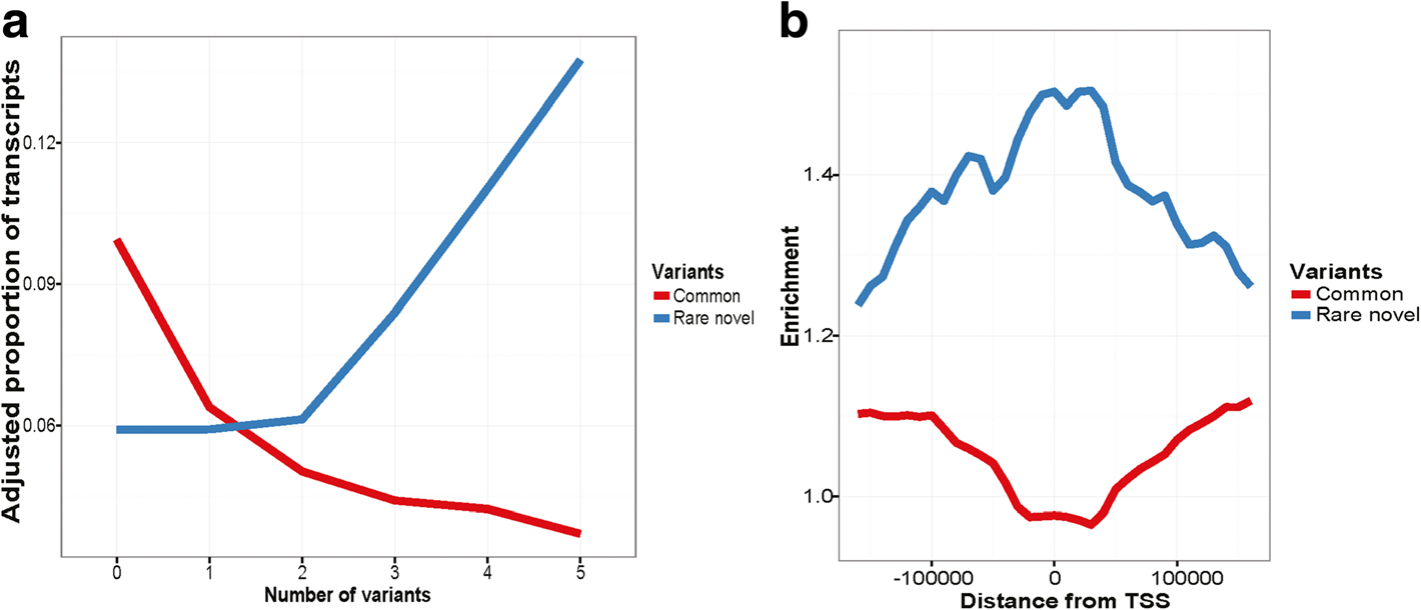
The number and location of rare and novel noncoding variants have an impact on gene. **a** Adjusted proportion of AI transcripts that contain 1 or more noncoding common (*red*) or rare and novel (*blue*) variants in transcripts vicinity (+/−20 kb from gene). Adjustment was based on average number of SNPs used to calculate ASE at each ASE levels. **b** Fold enrichment of common (*red*) or rare and novel (*blue*) variants in AI vs all transcripts measuring their distance from transcription start sites (TSS). Transcripts with *p* < 0.05 were used. Sliding window of 80 kb every 10 kb was used

Having the advantage of higher power using this larger cohort, we observed that the more rare or novel variants there are within the vicinity of the transcribed region of a gene, the higher the likelihood is that the transcripts will display AI (Fig. 4a), which is not observed for common variants. Finally, we looked at the enrichment of rare or common variants around the TSS of transcripts with homozygous eSNP and observed a higher enrichment at +/− 50 kb from the TSS for rare variants compared to common variants (Fig. 4b).

Taken together we have shown that, rare and novel variants identified in human immune cells using the Immunoseq capture panel are enriched in DHSs that are highly correlated to the promoters of transcripts and in the coding regions of highly differentially expressed transcripts, for which the top associated SNP is homozygous. We also observed enrichment of rare and novel variants in the vicinity of the TSS regions, and the more rare or novel variants there are, the stronger is the allelic imbalance of the gene expression.

## Discussion

In this study, we used existing DHS mapping data to build a custom capture panel designed to enable efficient re-sequencing of key immune cell regulatory regions. Our “Immunoseq” panel provides the means to comprehensively assess both coding and non-coding variation that could be implicated in the development of immune and inflammatory diseases. Because the method is based on sequencing rather than genotyping it allows direct cost effective assessment of both rare and common variation without any reliance on LD or the need for imputation. We have shown that with high sequencing coverage we are able to study novel non-coding variants in a confident way, which cannot be realized using whole exome sequencing, or would be prohibitively expensive using whole genome sequencing (WGS). The targeted regions included in the Immunoseq panel overlap with GWAS hits in immune and inflammatory diseases and eQTLs of immune cells.

An inevitable drawback of the Immunoseq design is its inability to capture variants of relevance to the disease of interest that map outside the targeted regions. This limitation is illustrated by disease-associated SNPs that are not included in the panel. Since the Immunoseq panel was based exclusively on DHSs seen in immune cells, these missing associated SNPs could reflect regulatory effects that are associated with non-immune cell based aspects of the disease [10], e.g. gastrointestinal tract DHSs in ulcerative colitis. The fact that our panel captures the majority of the known immune and chronic inflammatory disease associated SNPs indicates that it will have broad utility across multiple immune related diseases.

Until now targeted capture methods have focused almost exclusively on the coding regions of the genome [43], which means that the effects of rare non-coding variants have largely been ignored in the analysis of complex traits. In our exploration of the approach we found that the non-coding rare and novel variants identified by Immunoseq frequently modify transcription factor binding motifs and show higher levels of selective constraint than are seen in included common sequence variants. This difference is expected based on evolutionary and population genetics principles, with common variants expected to be more neutral than rare ones [44].

A further novel aspect of the Immunoseq approach is its inherent ability to utilise ASE information to interrogate the functional impact of sequence variants on gene expression. The greater power of ASE allowed us to observe functional effects using a lower sample size of unrelated subjects than traditional eQTL analysis [45]. In total the rare and novel variants identified by Immunoseq explained 14 % of the residual allelic imbalance in expression observed amongst individuals homozygous for common variants know to influence ASE, indicating that rare and novel variants likely account for at least part of the AI observed in the transcripts from individuals heterozygous for common eSNPs. Comparing non-coding variants in DHSs to variants in coding exons, the coding variants appeared to have a stronger effect on gene expression. However, the opposite situation was observed for variants located in DHSs that are correlated with gene promoters, where the effect of the non-coding variants was larger than those of coding ones. Rare and novel variants with substantial effects on AI in particular genes may contribute to certain disease phenotypes. In contrast to previous studies, we did not limit our exploration to extreme phenotypes, but instead we investigated the whole spectrum of AI. In doing so, we observed that the effect of rare and novel variants on gene expression does not appear to be limited to extreme differences in allelic expression, but may also affect genes with moderate AI.

One further limitation of the study may be that not all transcripts for which the allelic expression is skewed were accounted for by rare variants identified by Immunoseq. Some variants exerting long range or trans effects will inevitably have been missed by not performing WGS. Nevertheless, as opposed to earlier studies [22, 23], we expand the exploration of rare variant effects to distal regulatory sites with correlated activity with gene promoter. While distal sites show enrichment, the strongest effect of rare and novel variants is found around the TSS of genes displaying AI. This observation indicates that variants can be clustered to perform collapsing association test for complex traits, which will permit the identification of rare and novel trait-associated variants and to easy linking of the variants to a specific gene.

## Conclusion

In this study, we show that targeted re-sequencing of cell specific active regulatory regions can be an efficient means to identify functionally relevant variation that is considerably more cost effective than WGS. Immunoseq provides an efficient means to identify rare and novel, coding and non-coding variation of relevance in complex traits involving the immune system and to study the impact of rare and novel non-coding regulatory variants on other epigenetic traits.

## Additional files

Additional file 1: Table S1. Cell type selected to target regulatory regions in immune cells. **Table S2.** Cell types selected to target regulatory regions in other cell types not related to immune function. **Table S3.** Summary of shared common, rare and novel variants in selected DHS regions of different immune cells. **Table S4.** Sequencing statistics of the Cambridge Multiple sclerosis samples with Immunoseq. **Figure S1.** Variants quality control. **Figure S2.** Comparing sequencing data for NA18502 sample (Complete Genomics data and Immunoseq) considering only heterozygous SNVs identified by Complete Genomics that fall within Immunoseq custom capture panel regions. **Figure S3.** ImmunoChip hits that falls into Immunoseq custom capture panel. **Figure S4.** Discovery set distribution of allele specific expression (ASE). **Figure S5.** Average number of SNPs used to calculate allele specific expression (ASE) in discovery set samples. **Figure S6.** Adjusted proportion of transcripts with common (red), rare (blue) or novel (green) noncoding variants in the vicinity (+/-20kb) of a gene based on different allelic imbalance: 1.5 to 9, 2 to 9, 2.5 to 9, 3 to 9 and 3.5 to 9 fold difference in the discovery set. **Figure S7.** Discovery set distribution of Allelic imbalance (AI). **Figure S8.** Enrichment of proportion of AI transcripts with rare or novel variants in vicinity of a gene compared to AI transcripts with common variants in vicinity of a gene in the discovery set. **Figure S9.** Fold difference between proportions of AI transcripts with rare or novel variants in vicinity compared to AI transcripts with common variants in vicinity in the discovery set. **Figure S10.** Enrichment between proportions of AI transcripts with rare or novel variants in vicinity compared to AI transcripts with common variants in vicinity in the discovery set. **Figure S11.** Replication set distribution of allele specific expression (ASE). **Figure S12.** Average number of SNPs used to calculate allele specific expression (ASE) in the replication set. **Figure S13.** Distribution of allele specific expression of all transcripts and transcripts that did not carry the common allele in a heterozygous state. **Figure S14.** Replication set distribution of Allelic Imbalance (AI). **Figure S15.** Enrichment between proportions of AI transcripts with rare or novel variants in vicinity compared to AI transcripts with common variants in vicinity in the discovery and replication set. **Figure S16.** Enrichment between proportions of AI transcripts with rare or novel variants in vicinity compared to AI transcripts with common variants in vicinity in the replication set. (DOCX 1.61 mb) Additional file 2: Table S5. Rare and novel variants identified using Immunoseq and DHS cell-type they fall into. (XLSX 2.46 mb)

## Abbreviations

AI: Allelic imbalance
ASE: Allele-specific expression
CADD: Combined annotation dependent depletion
DHS: DNase I hypersensitive sites
DNase I: Deoxyribonuclease I
DNase-seq: DNase I sequencing
eQTL: Expression quantitative trait loci
eSNP: SNPs altering expression
FIMO: Finding Individual Motif Occurrence
GERP: Genomic Evolutionary Rate Profiling
GWAS: Genome-wide association study
HLA: Human leucocyte antigen
MAF: Minor allele frequency
MHC: Major histocompatibility complex
NGS: Next-generation sequencing
RNA-seq: RNA sequencing
SNP: Single nucleotide polymorphism
TSS: Transcription start site
WES: Whole-exome sequencing
WGS: Whole-genome sequencing
