# Supplementary material for: Immunoseq: the identification of functionally relevant variants through targeted capture and sequencing of active regulatory regions in human immune cells
Source: BMC Med Genomics. 2016 Sep 13;9(1):59. doi: 10.1186/s12920-016-0220-7 (PMC5022205; doi:10.1186/s12920-016-0220-7)
Supplement: Additional file 1: Table S1. — Cell type selected to target regulatory regions in immune cells. Table S2. Cell types selected to target regulatory regions in other cell types not related to immune function. Table S3. Summary of shared common, rare and novel variants in selected DHS regions of different immune cells. Table S4. Sequencing statistics of the Cambridge Multiple sclerosis samples with Immunoseq. Figure S1. Variants quality control. Figure S2. Comparing sequencing data for NA18502 sample (Complete Genomics data and Immunoseq) considering only heterozygous SNVs identified by Complete Genomics that fall within Immunoseq custom capture panel regions. Figure S3. ImmunoChip hits that falls into Immunoseq custom capture panel. Figure S4. Discovery set distribution of allele specific expression (ASE). Figure S5. Average number of SNPs used to calculate allele specific expression (ASE) in discovery set samples. Figure S6. Adjusted proportion of transcripts with common (red), rare (blue) or novel (green) noncoding variants in the vicinity (+/-20kb) of a gene based on different allelic imbalance: 1.5 to 9, 2 to 9, 2.5 to 9, 3 to 9 and 3.5 to 9 fold difference in the discovery set. Figure S7. Discovery set distribution of Allelic imbalance (AI). Figure S8. Enrichment of proportion of AI transcripts with rare or novel variants in vicinity of a gene compared to AI transcripts with common variants in vicinity of a gene in the discovery set. Figure S9. Fold difference between proportions of AI transcripts with rare or novel variants in vicinity compared to AI transcripts with common variants in vicinity in the discovery set. Figure S10. Enrichment between proportions of AI transcripts with rare or novel variants in vicinity compared to AI transcripts with common variants in vicinity in the discovery set. Figure S11. Replication set distribution of allele specific expression (ASE). Figure S12. Average number of SNPs used to calculate allele specific expression (ASE) in the replication set. Figure S13. [file 12920_2016_220_MOESM1_ESM.docx]

**Additional file 1**

**Table S1.** Cell type selected to target regulatory regions in immune cells

| Cell types | Number of bins | Final number of bins | Number of samples | Accession number (GEO) |
| --- | --- | --- | --- | --- |
| CD3+ | 250,000 | 259,321 | 4 | GSM665837, GSM701488, GSM701516, GSM774201 |
| CD3+ cord blood | 150,000 | 97,243 | 2 | GSM701525, GSM701526 |
| CD4+ | 200,000 | 196,585 | 8 | GSM665812, GSM665839, GSM701489, GSM701491, GSM701539, GSM817166, wgEncodeUwDnaseCd4naivewb11970640AlnRep1, wgEncodeUwDnaseCd4naivewb78495824AlnRep1 |
| CD8+ | 250,000 | 229,426 | 5 | GSM665813, GSM665838, GSM701499, GSM701540, GSM817160 |
| CD14+ | 250,000 | 271,497 | 4 | GSM701503, GSM701541, wgEncodeUwDnaseMonocd14ro1746AlnRep1V2, wgEncodeUwDnaseMonocd14ro1746AlnRep2 |
| CD19+ | 250,000 | 240,941 | 3 | GSM701492, GSM701493, GSM701507 |
| CD20+ | 225,000 | 200,000 | 1 | GSM701500 |
| CD34+ | 250,000 | 242,494 | 14 | GSM493384, GSM493386, GSM493387, GSM530652, GSM530657, GSM530658, GSM530659, GSM530660, GSM530663, GSM530664, GSM595914, GSM595917, GSM595918, GSM595919 |
| CD56+ | 200,000 | 183,311 | 3 | GSM665820, GSM665836, GSM701508 |
| Th1 | 100,000 | 64,012 | 2 | wgEncodeOpenChromDnaseAdultcd4th1AlnRep1, wgEncodeOpenChromDnaseAdultcd4th1AlnRep2 |
| Th2 | 300,000 | 291,859 | 1 | GSM736502 |
| Th17 | 100,000 | 100,000 | 1 | wgEncodeUwDnaseTh17AlnRep1 (to verify) |

**Table S2.** Cell types selected to target regulatory regions in other cell types not related to immune function

| Cell types/Tissue | Number of bins | Final number of bins | Number of samples | Accession number (GEO) |
| --- | --- | --- | --- | --- |
| Fetal Lung | 250,000 | 231,527 | 11 | GSM530662, GSM595915, GSM595916,  GSM595921, GSM595924, GSM595925, GSM595927, GSM595929, GSM595930, GSM665805, GSM665806 |
| Fetal Kidney | 200,000 | 193,630 | 6 | GSM493385, GSM774221, GSM817159, GSM878666, GSM1024608, GSM1027329 |
| Fetal Brain | 200,000 | 173,884 | 9 | GSM530651, GSM595913, GSM595920, GSM595922, GSM595923, GSM595926, GSM595928, GSM665804, GSM1027328 |
| Fetal Small intestine | 250,000 | 225,102 | 11 | GSM665825, GSM665835, GSM701487, GSM701496, GSM701504, GSM701530, GSM774205, GSM774210, GSM774216, GSM817161, GSM817187 |
| Fetal Large intestine | 250,000 | 233,707 | 9 | GSM701490, GSM701495, GSM701531, GSM774213, GSM774214, GSM774217, GSM774220, GSM817162, GSM817188 |
| Fetal Renal cortex | 250,000 | 245,861 | 10 | GSM701494, GSM701502, GSM701529, GSM701532, GSM817176, GSM878629, GSM878667, GSM1027314, GSM1027316, GSM1027323 |
| Fetal Stomach | 250,000 | 232,109 | 13 | GSM701498, GSM701521, GSM701528, GSM701538, GSM774202, GSM774212, GSM817173, GSM817199, GSM878660, GSM878665, GSM1024606, GSM1027318, GSM1027331 |
| Fetal Arm muscle | 250,000 | 241,617 | 15 | GSM701506, GSM701535, GSM774223, GSM774239, GSM817178, GSM817184, GSM817214, GSM817216, GSM878610, GSM878618, GSM878619, GSM878620, GSM878625, GSM878638, GSM1024605 |
| Fetal Placenta | 250,000 | 231,726 | 5 | GSM774215, GSM774219, GSM817219, GSM878659, GSM1027343 |
| Fetal Adrenal gland | 200,000 | 179,763 | 5 | GSM817165, GSM817167, GSM878658, GSM1027310, GSM1027311 |
| Fetal Testis | 250,000 | 198,545 | 2 | GSM878617, GSM1027319 |
| Fetal Ovary | 100,000 | 100,000 | 1 | GSM1027306 |

**Table S3.** Summary of shared common, rare and novel variants in selected DHS regions of different immune cells

| **Common/**  **Rare/ novel** | **CD3+** | **CD3+ cord blood** | **CD4+** | **CD8+** | **CD14+** | **CD19+** | **CD20+** | **CD34+** | **CD56+** | **Th1** | **Th2** | **Th17** |
| --- | --- | --- | --- | --- | --- | --- | --- | --- | --- | --- | --- | --- |
| **CD3+** | 54,570/ 9,834/ 5,774 |  |  |  |  |  |  |  |  |  |  |  |
| **CD3+ cord blood** | 21,628/ 3,880/ 2,469 | 21,730/ 3,909/ 2,481 |  |  |  |  |  |  |  |  |  |  |
| **CD4+** | 40,663/ 7,288/ 4,416 | 21,421/ 3,849/ 2,436 | 42,354/ 7,520/ 4,561 |  |  |  |  |  |  |  |  |  |
| **CD8+** | 45,291/ 7,995/ 4,885 | 21,385/ 3,827/ 2,442 | 38,704/ 6,840/ 4,193 | 49,086/ 8,602/ 5,239 |  |  |  |  |  |  |  |  |
| **CD14+** | 26,697/ 4,973/ 3,062 | 18,040/ 3,328/ 2,083 | 25,084/ 4,585/ 2,819 | 25,886/ 4,746/ 2,948 | 59,581/ 9,829/ 5,819 |  |  |  |  |  |  |  |
| **CD19+** | 35,476/ 6,364/ 3,992 | 21,245/ 3,822/ 2,428 | 32,055/ 5,731/ 3,596 | 34,235/ 6,110/ 3,862 | 27,704/ 5,087/ 3,150 | 50,942/ 8,773/ 5,407 |  |  |  |  |  |  |
| **CD20+** | 20,035/ 3,654/ 2,256 | 14,707/ 2,657/ 1,677 | 18,817/ 3,448/ 2,127 | 19,488/ 3,519/ 2,172 | 18,709/ 3,385/ 2,089 | 19,000/ 3,441/ 2,143 | 43,910/ 7,047/ 4,288 |  |  |  |  |  |
| **CD34+** | 31,128/ 5,728/ 3,548 | 19,886/ 3,586/ 2,295 | 28,223/ 5,149/ 3,138 | 29,834/ 5,437/ 3,408 | 31,088/ 5,544/ 3,461 | 29,577/ 5,366/ 3,402 | 20,419/ 3,654/ 2,276 | 53,076/ 9,165/ 5,552 |  |  |  |  |
| **CD56+** | 35,904/ 6,434/ 3,980 | 20,571/ 3,684/ 2,360 | 32,535/ 5,783/ 3,633 | 35,943/ 6,387/ 3,975 | 24,905/ 4,550/ 2,787 | 29,661/ 5,290/ 3,371 | 18,076/ 3,343/ 2,064 | 27,573/ 5,033/ 3,138 | 40,046/ 7,156/ 4,359 |  |  |  |
| **Th1** | 12,015/ 2,171/ 1,304 | 9,770/ 1,714/ 1,082 | 11,479/ 2,062/ 1,265 | 11,846/ 2,119/ 1,280 | 10,734/ 1,930/ 1,179 | 11,060/ 1,997/ 1,224 | 9,063/ 1,638/ 990 | 11,365/ 2,044/ 1,252 | 11,181/ 2,001/ 1,232 | 13,025/ 2,412/ 1,414 |  |  |
| **Th2** | 36,811/ 6,280/ 3,903 | 18,595/ 3,285/ 2,132 | 32,002/ 5,456/ 3,451 | 34,935/ 5,876/ 3,742 | 24,458/ 4,337/ 2,716 | 30,582/ 5,220/ 2,392 | 18,023/ 3,166/ 1,964 | 27,643/ 4,895/ 3,096 | 29,154/ 5,018/ 3,182 | 11,768/ 2,043/ 1,250 | 59,458/ 9,775/ 5,707 |  |
| **Th17** | 31,088/ 3,260/ 1,979 | 11,617/ 2,254/ 1,443 | 15,581/ 3,010/ 1,847 | 16,234/ 3,076/ 1,894 | 13,322/ 2,665/ 1,589 | 13,051/ 2,581/ 1,596 | 9,967/ 1,949/ 1,207 | 14,468/ 2,808/ 1,713 | 15,758/ 2,955/ 1,867 | 7,574/ 1,417/ 907 | 17,141/ 3,186/ 1,922 | 22,026/ 4,146/ 2,366 |

**Table S4.** Sequencing statistics of the Cambridge Multiple sclerosis samples with Immunoseq

|  | Mean target coverage | Bases on target (%)^1^ | Target region without coverage (%)^2^ | Target bases with >=10x coverage (%)^3^ | Sequencing platform | Level of multiplexing |
| --- | --- | --- | --- | --- | --- | --- |
| Cambridge Multiple Sclerosis cohort and healthy controls (n=180) | 31X | 68.76 | 1.9 | 73 | HiSeq2000 | 6X |

Alignment to the human hg19 reference genome, and variant calling (HaplotypeCaller) to identify all SNPs were performed. Shows average values across samples. ^1^ On and near bait bases/good quality bases aligned (according to Picards metrics). ^2^The percentage of target region that did not reach 2x coverage over any base.^3^ The percentage of all target bases achieving 10X or higher coverage. We considered a variant to be true at >=10 depth.

| A) | B) | C) |
| --- | --- | --- |
| 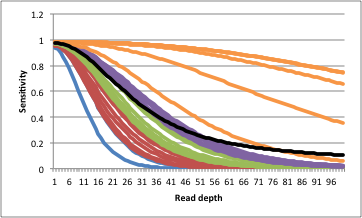 | 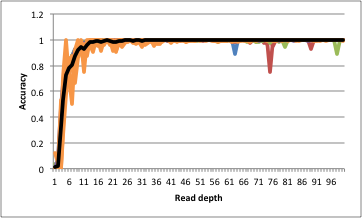 | 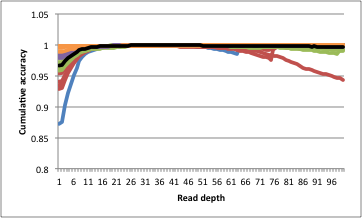 |
| 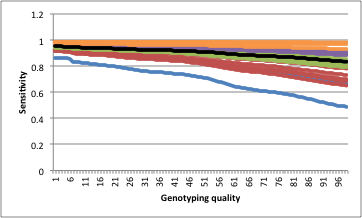 | 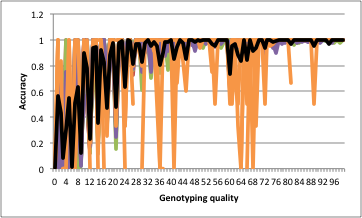 | 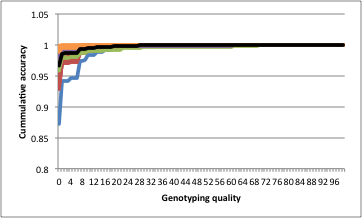 |
| 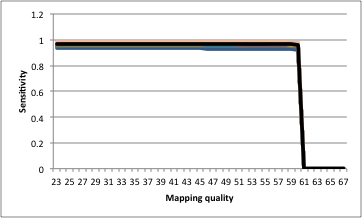 | 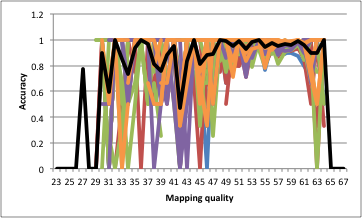 |  |
| **Figure S1. Variants quality control.** Comparing sequencing data to genotyping data (Human Omni2.5 BeadChip) considering only heterozygous SNPs for the discovery set. Average is shown in black, samples are coloured according to mean coverage: blue=15-20, red=20-30, green=30-40, purple= 40-50, orange>=50. **A**) Sensitivity: number of genotyped SNPs that are called in the Immunoseq at increasing read depth/genotyping quality/mapping quality. **B)** Accuracy: proportion of variants captured at each read depth/genotyping quality/mapping quality matching genotyping data. **C)** Cumulative accuracy: proportion of variants captured at each read depth/genotyping quality and over, matching genotyping data. | | |


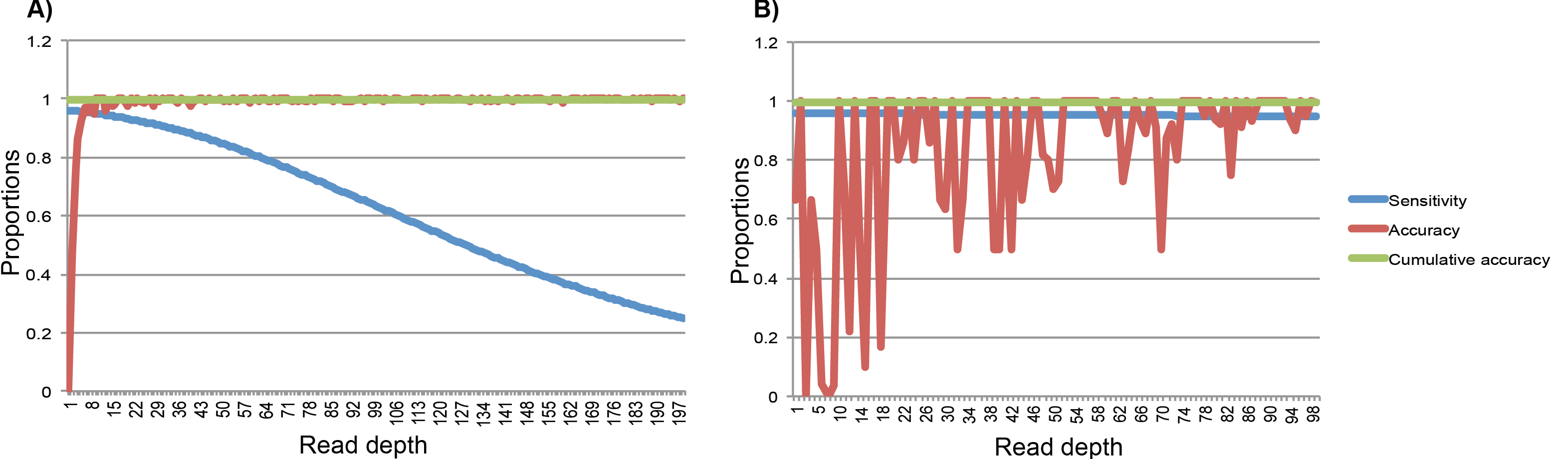


**Figure S2. Comparing sequencing data for NA18502 sample** (Complete Genomics data and Immunoseq) considering only heterozygous SNVs identified by Complete Genomics that fall within Immunoseq custom capture panel regions. Sensitivity: number of genotyped SNPs observed in the Immunoseq at increasing read depth/genotype quality (ex: over 10 read depth we capture 95% of the heterozygous variants). Accuracy: proportion of variants captured at each depth (and over) matching Complete Genomics genotype calls. **A)** Read depth. **B)** Genotyping quality.

| 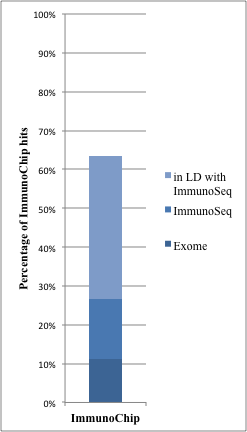 |  |
| --- | --- |
| **Figure S3. ImmunoChip hits that falls into Immunoseq custom capture panel. A)** ImmunoChip hits that fall within Immunoseq capture panel regions. (Cut-off of 1x10^-8^ was used to select hits to analyze, SNPs in LD selected based on r^2^>0.9, HLA hits and region as well as chromosome X SNPs were excluded from the analyses). SNP in LD = ImmunoChip hits that have a SNP in LD represented by Immunoseq. **B)** Enrichment of hits (same as in A) and proximal SNPs (LD r^2^>0.9) that fall in DHSs selected for immune cell types compared to DHSs selected from other tissues (either all or non-overlapping ones) and regions randomly selected (1000 times) from the whole genome (either entire genome or only non-coding excluding the HLA region). Significance was calculated using Fisher exact test. | |


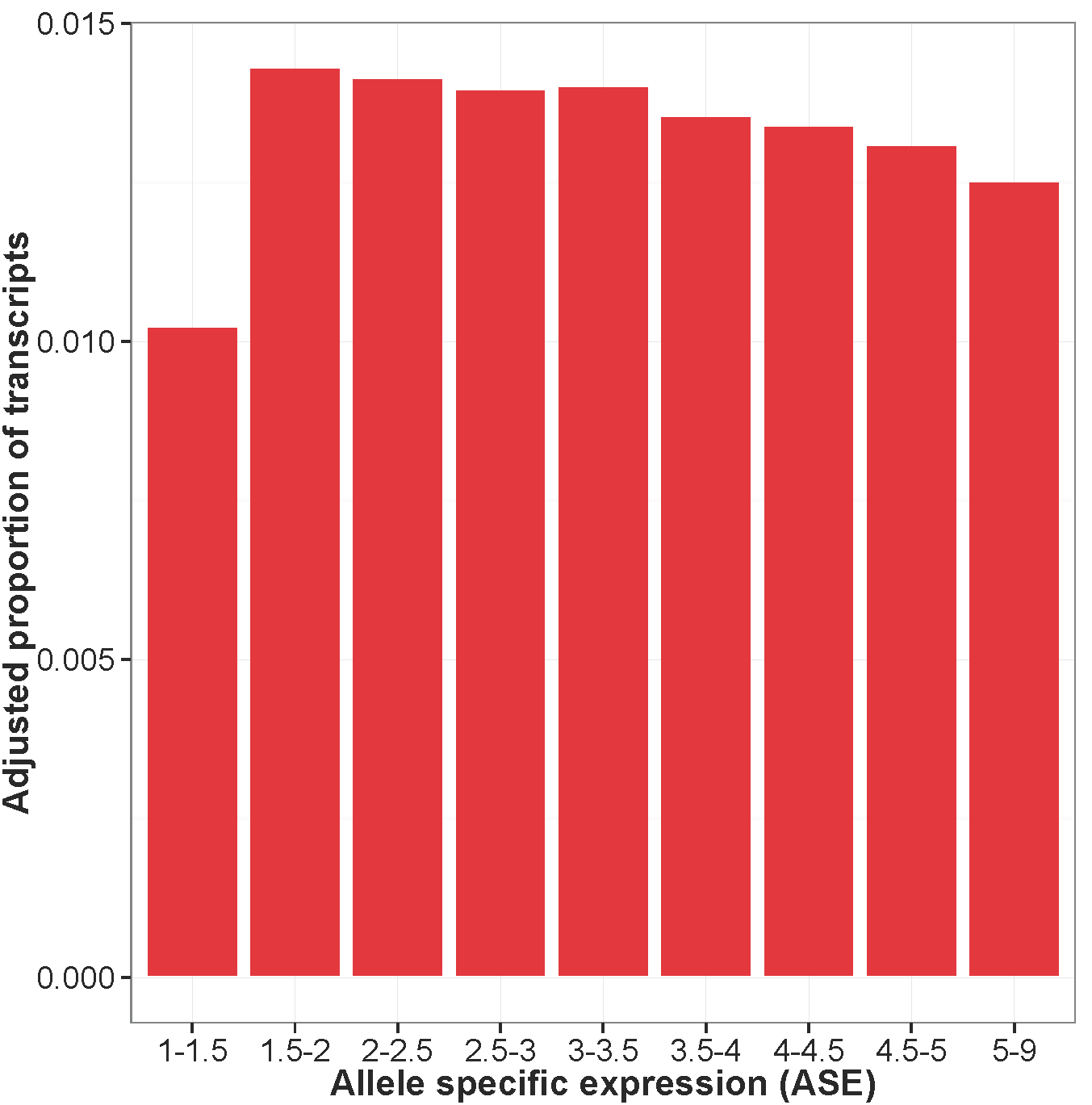

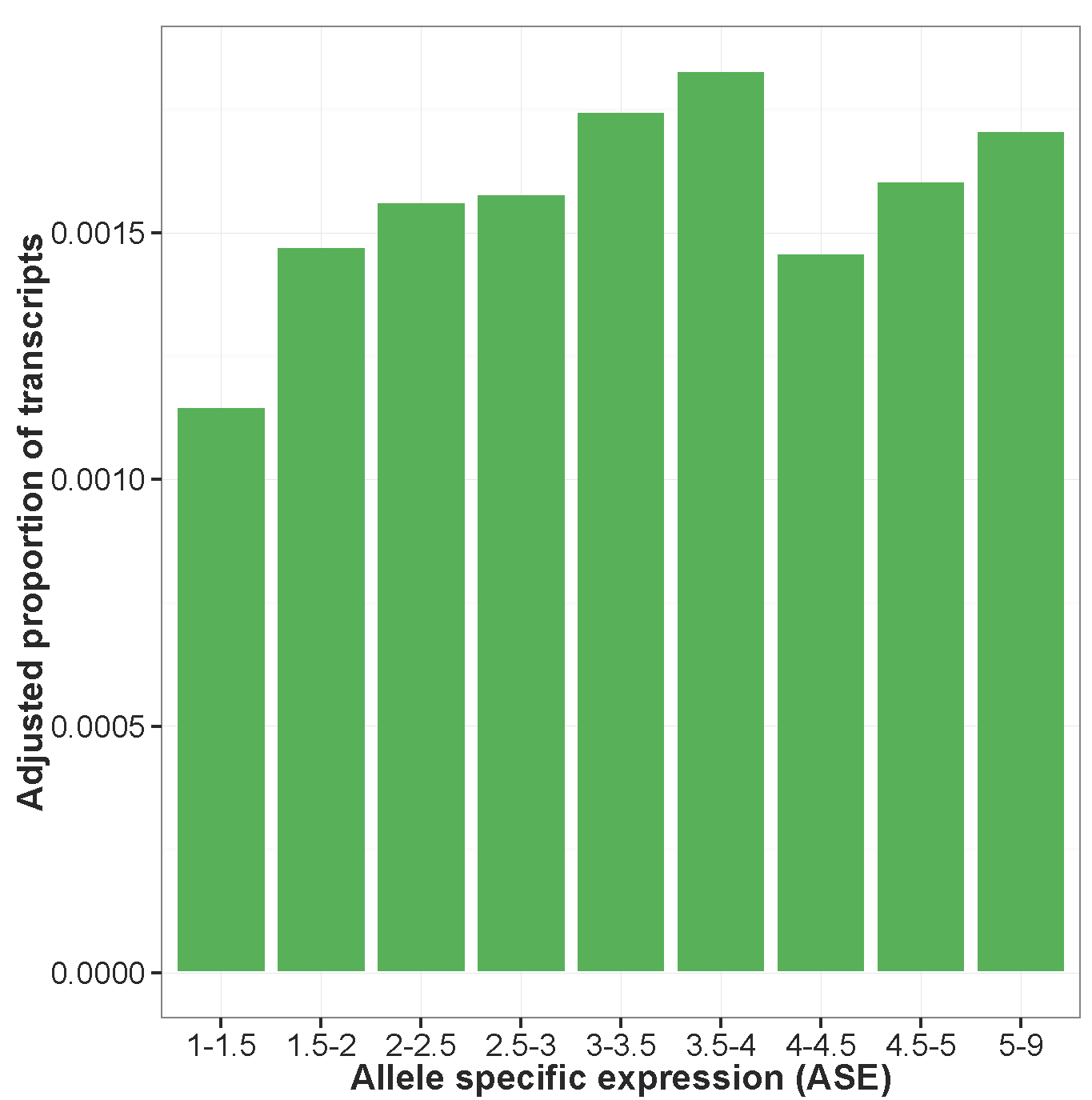

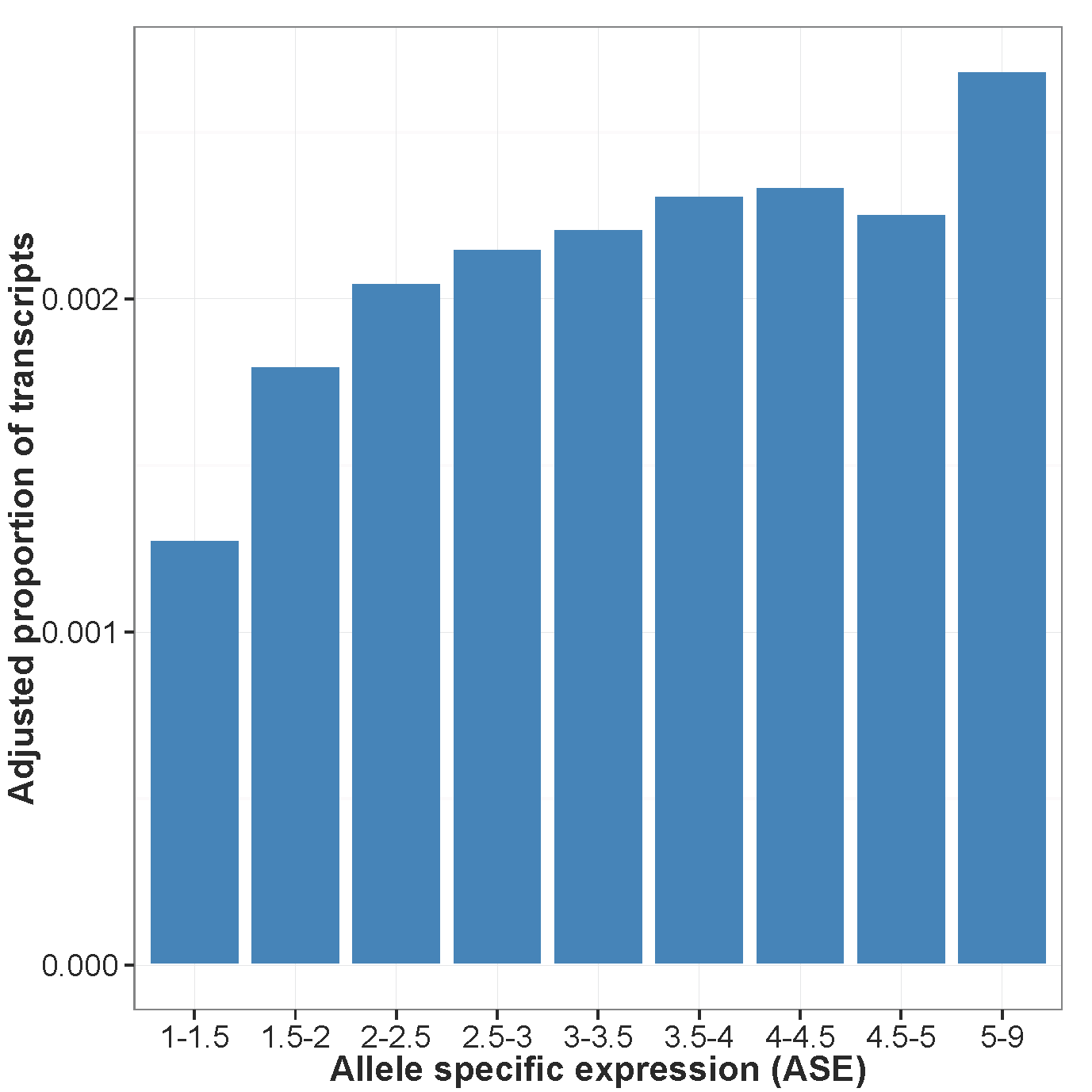


**Figure S4. Discovery set distribution of allele specific expression (ASE).** Distribution of proportion of ASE in transcripts with common (red), rare (blue) or novel (green) noncoding variants in vicinity (+/-20kb from gene) adjusted for average number of SNPs used to calculate ASE.

| 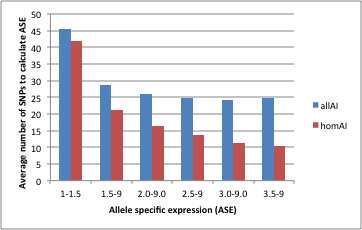 | 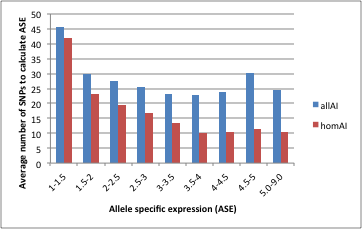 |
| --- | --- |
| **Figure S5. Average number of SNPs used to calculate allele specific expression (ASE) in discovery set samples**. Comparing all transcripts for which ASE was measured (allAI) and transcripts for which the top associated SNP that drives the association across samples is homozygous (homAI). | |


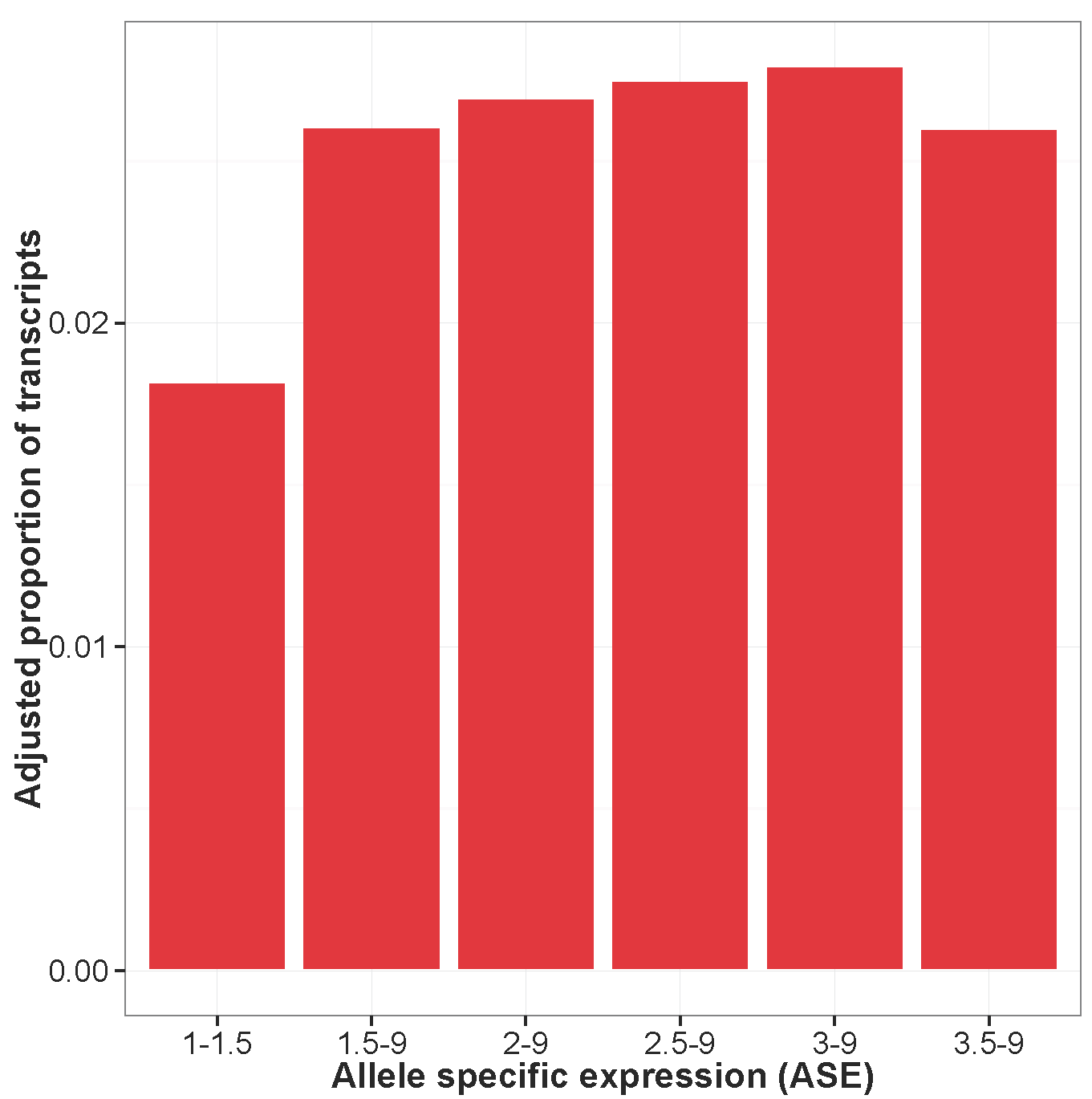

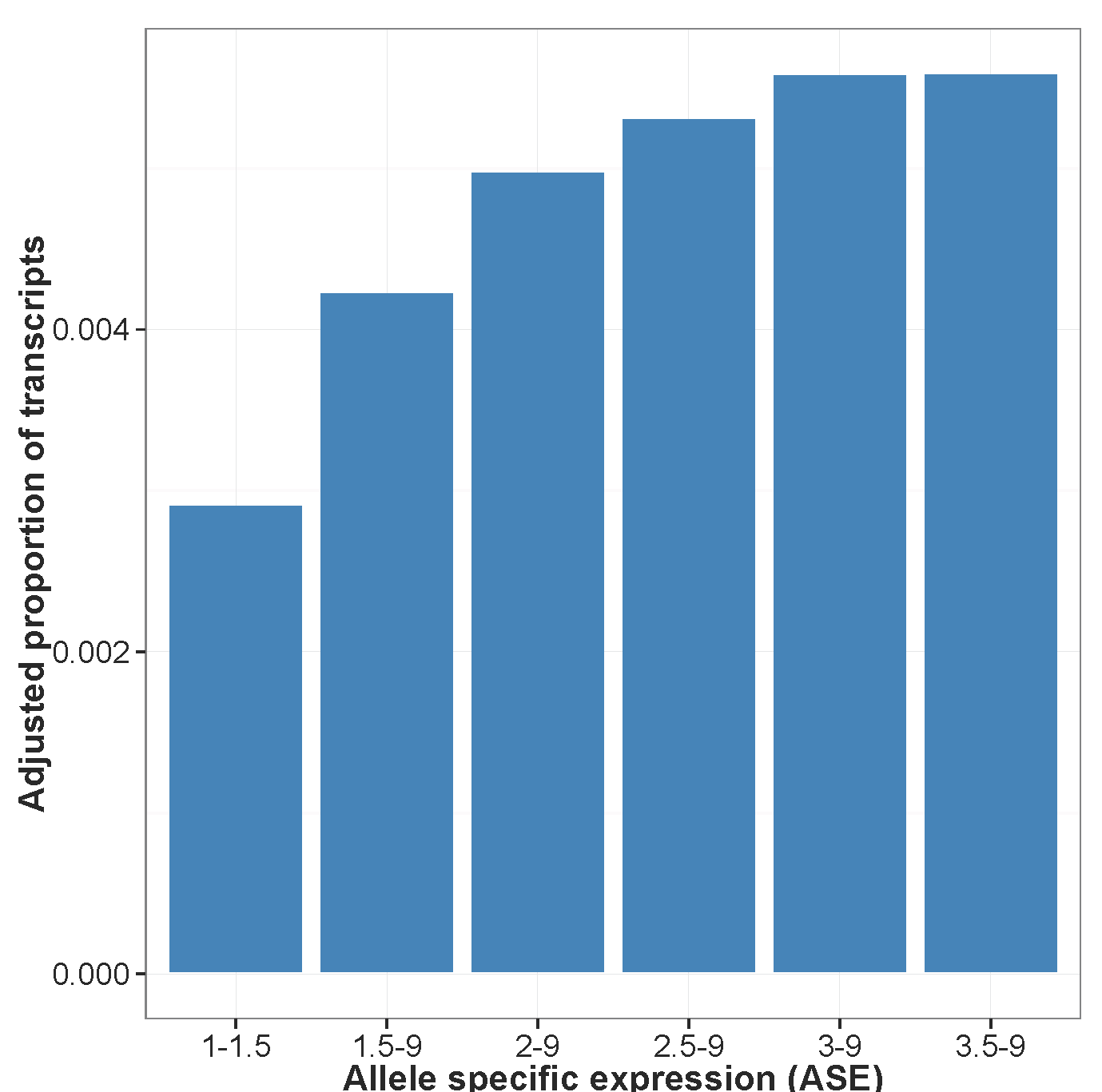

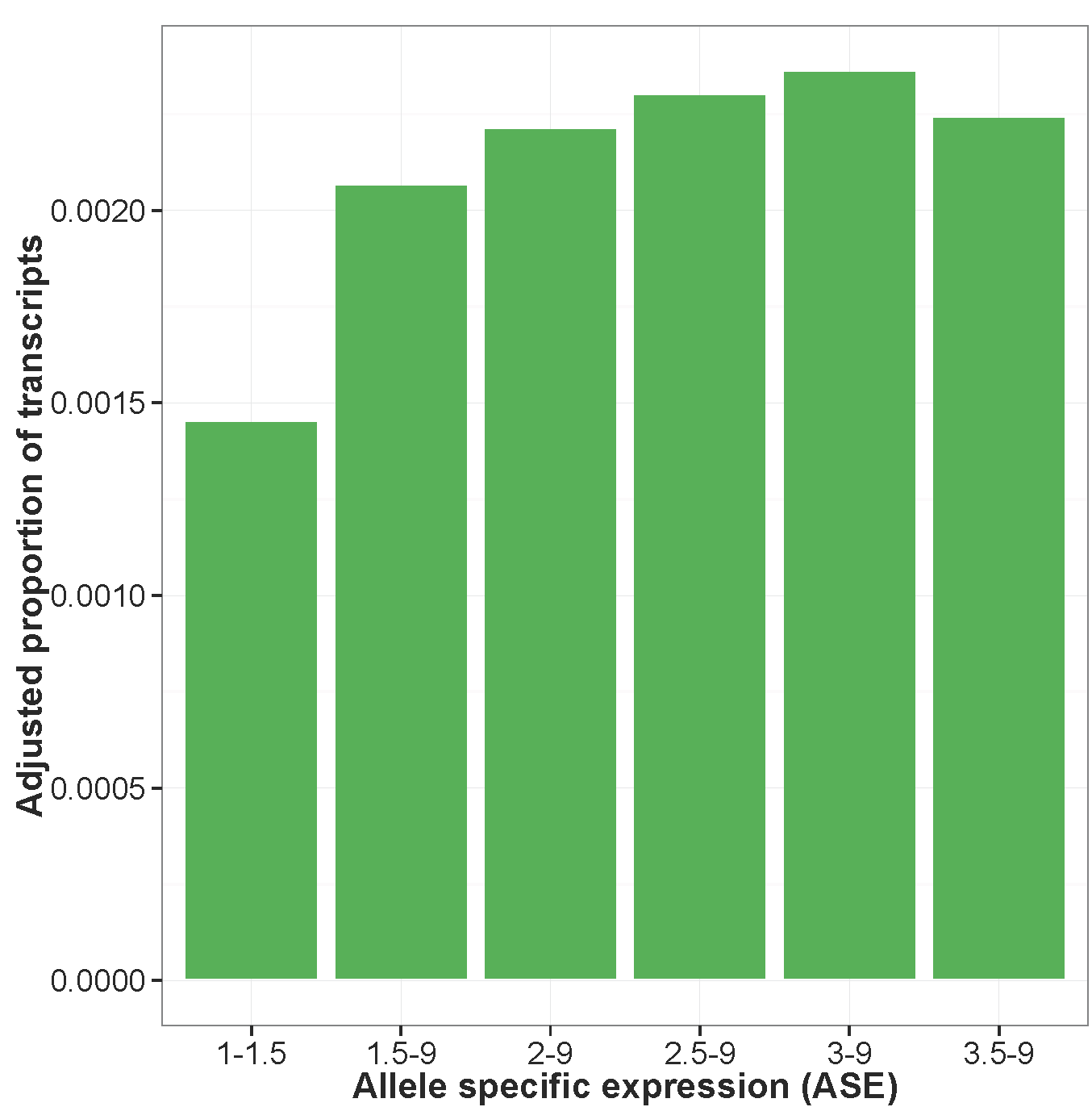


**Figure S6. Adjusted proportion of transcripts** with common (red), rare (blue) or novel (green) noncoding variants in the vicinity (+/-20kb) of a gene based on different allelic imbalance: 1.5 to 9, 2 to 9, 2.5 to 9, 3 to 9 and 3.5 to 9 fold difference in the discovery set. Adjustment was based on average number of SNPs used to calculate ASE at each ASE levels.


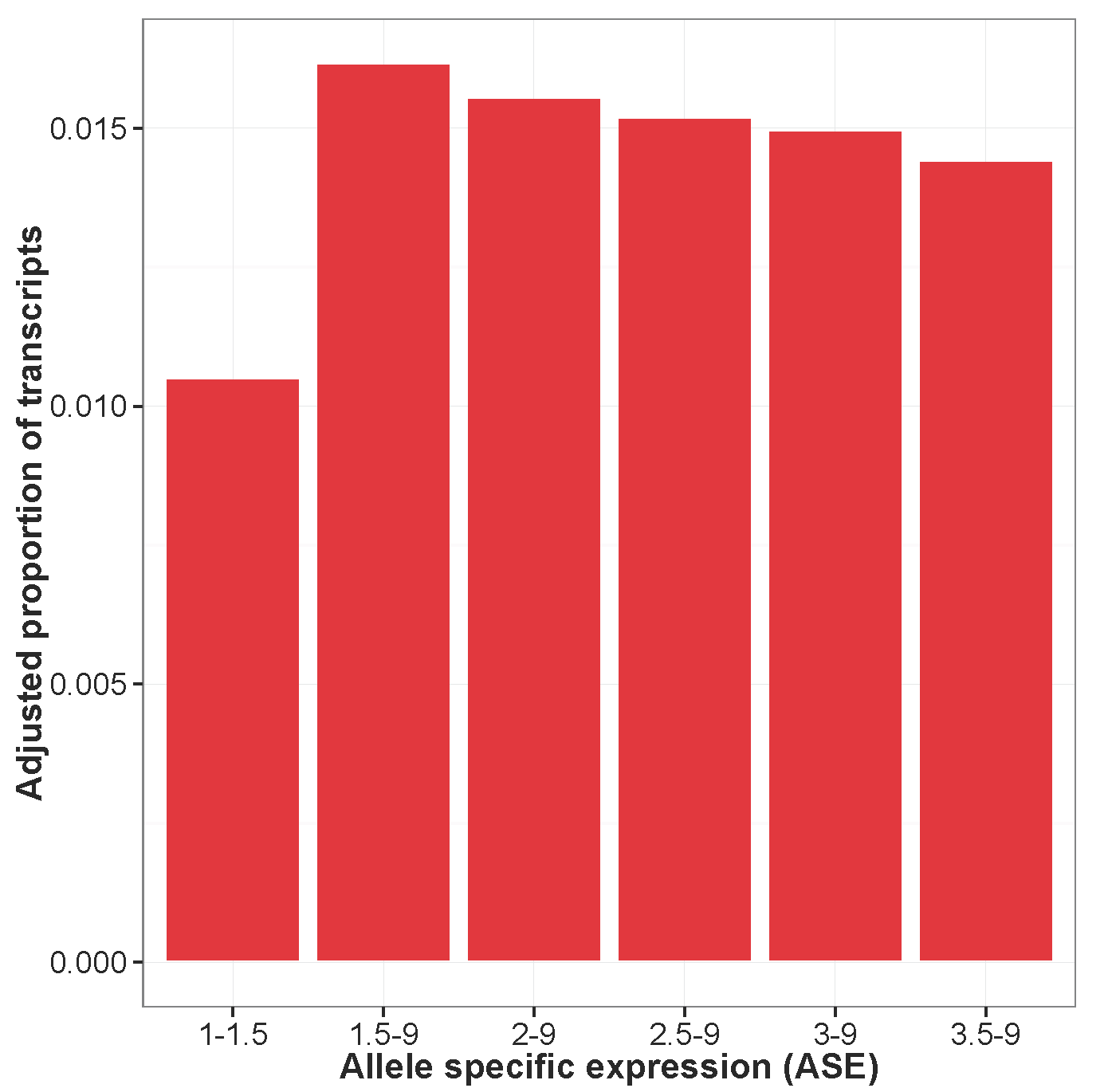

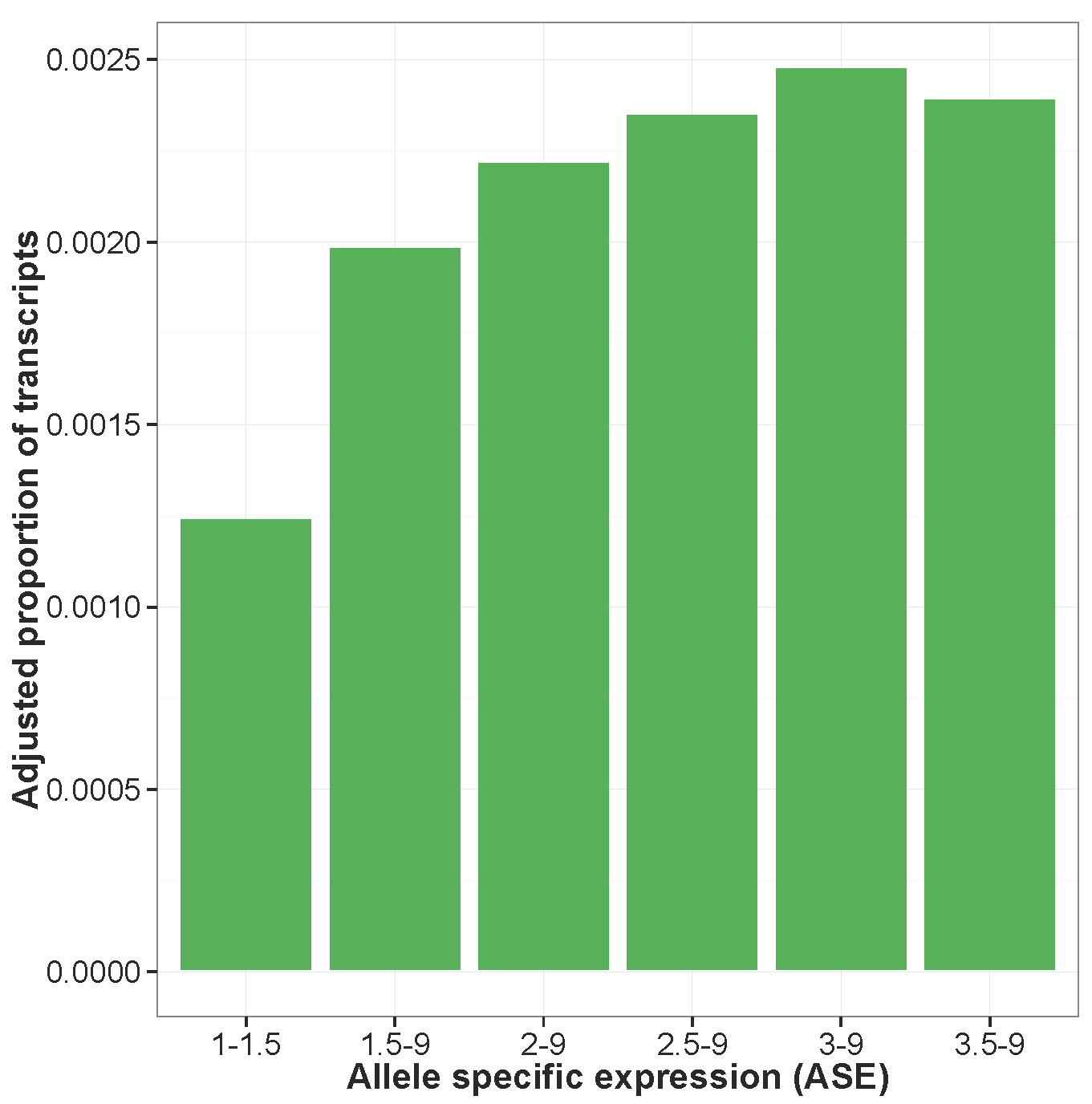

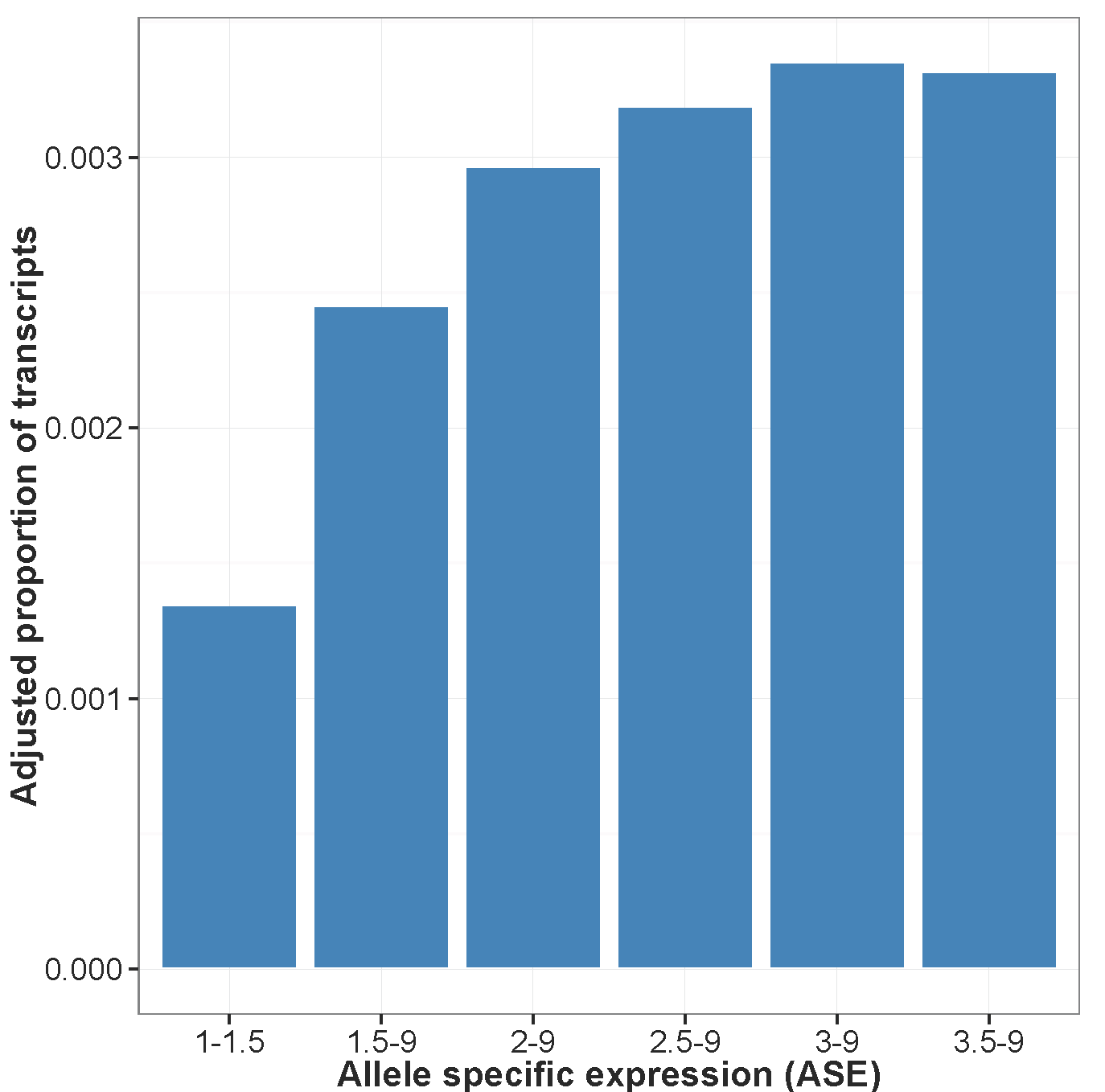


**Figure S7. Discovery set distribution of Allelic imbalance (AI).** Adjusted proportion of transcripts with common (red), rare (blue) or novel (green) noncoding variants in vicinity (+/-20kb from gene) based on different AI: 1.5 to 9, 2 to 9, 2.5 to 9, 3 to 9 and 3.5 to 9 fold difference. Only included transcripts for which the top associated SNP is homozygous in the sample (homAI).


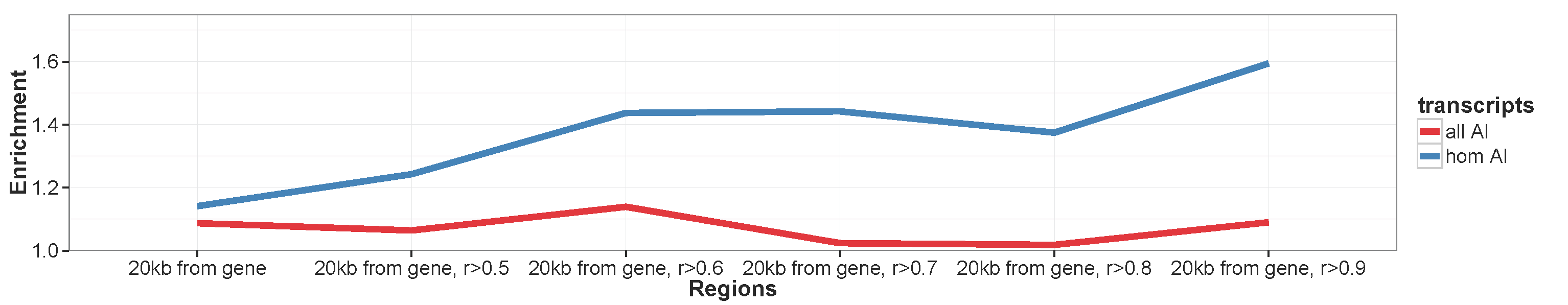

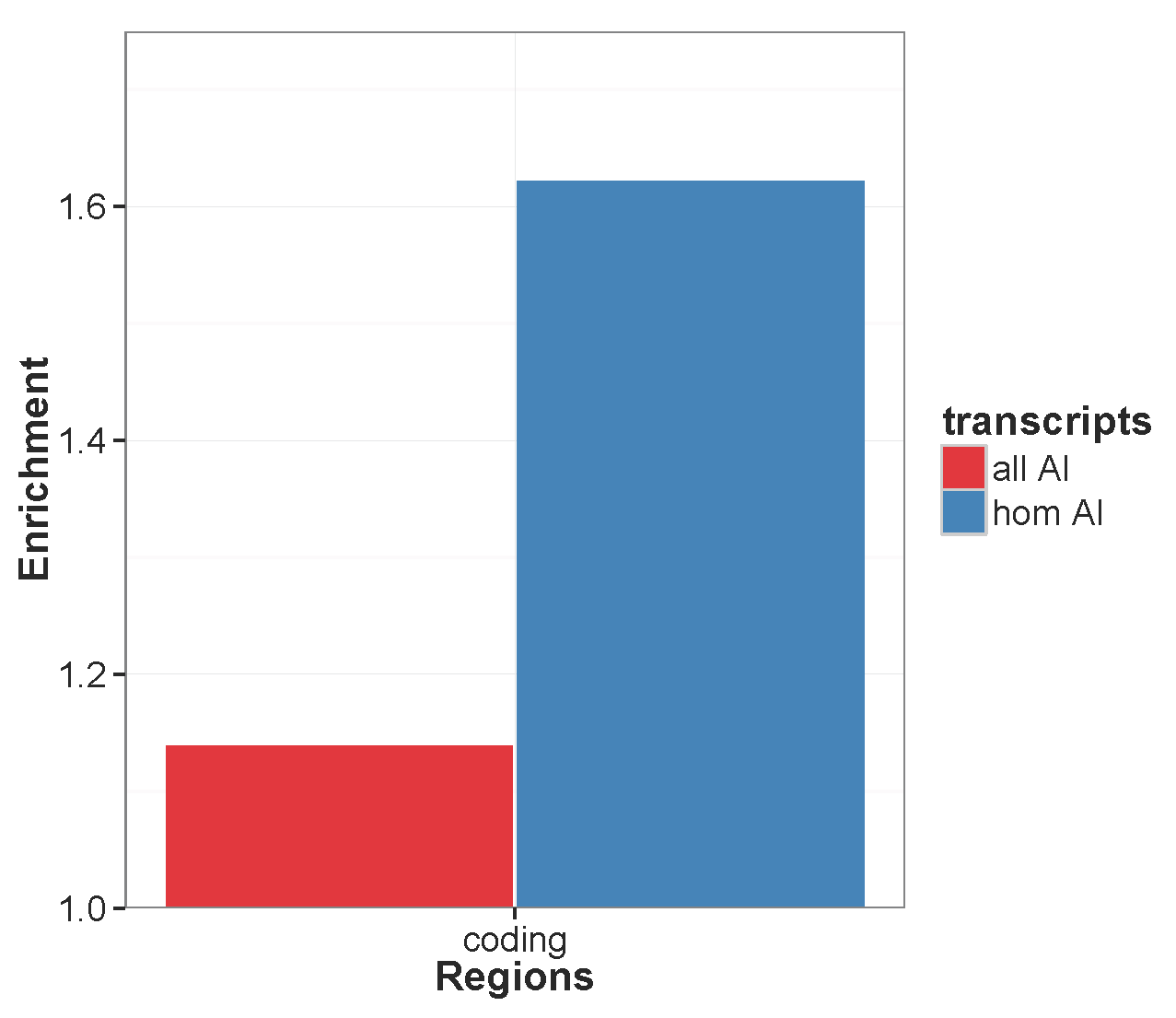


**Figure S8. Enrichment of proportion of AI transcripts with rare or novel variants in vicinity of a gene compared to AI transcripts with common variants in vicinity of a gene in the discovery set**. We looked at coding (histogram) vs noncoding variants as well as noncoding variants in DHS region correlated with the promoter (Pearson correlation r>0.5 to 0.9). Shown are all transcripts where allelic imbalance was measured (allAI, red) and transcripts for which the top associated SNP is homozygous in the sample (homAI, blue). Linear regression slope for homAI=0.076 (p-value= 0.018) and allAI= -0.007 (p-value= 0.591). Allelic imbalance genes are considered as >=2 fold between the alleles and equally expressed genes are <=1.5 fold.


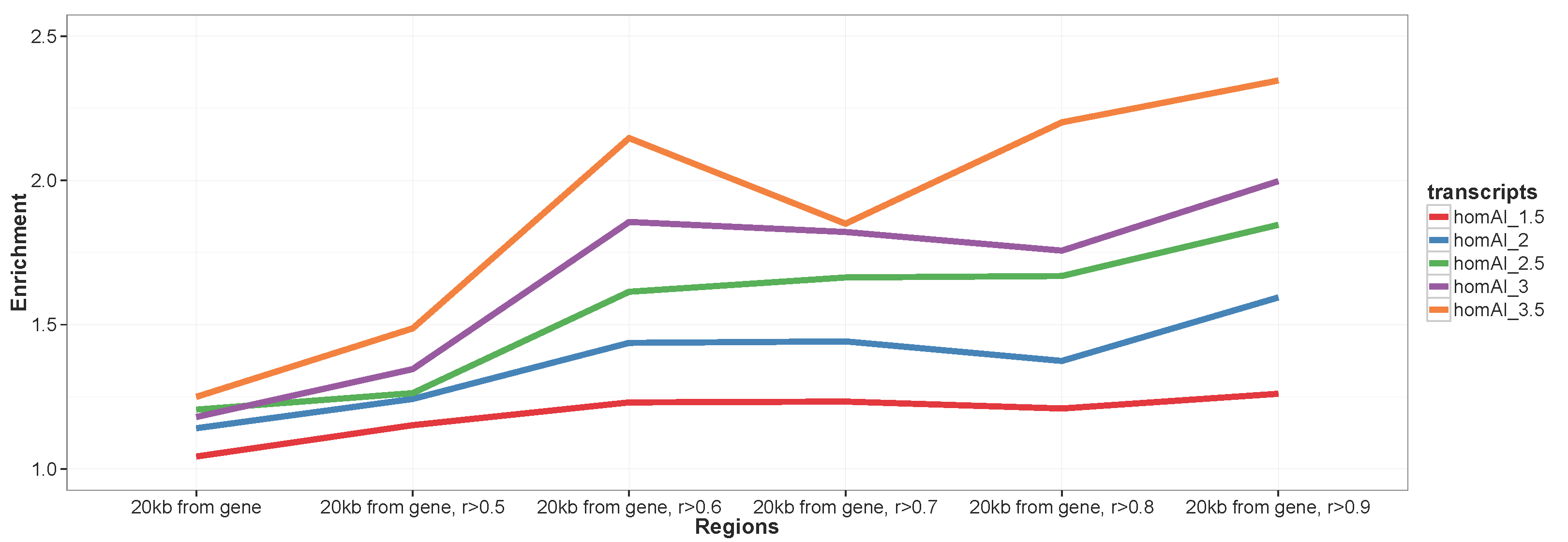

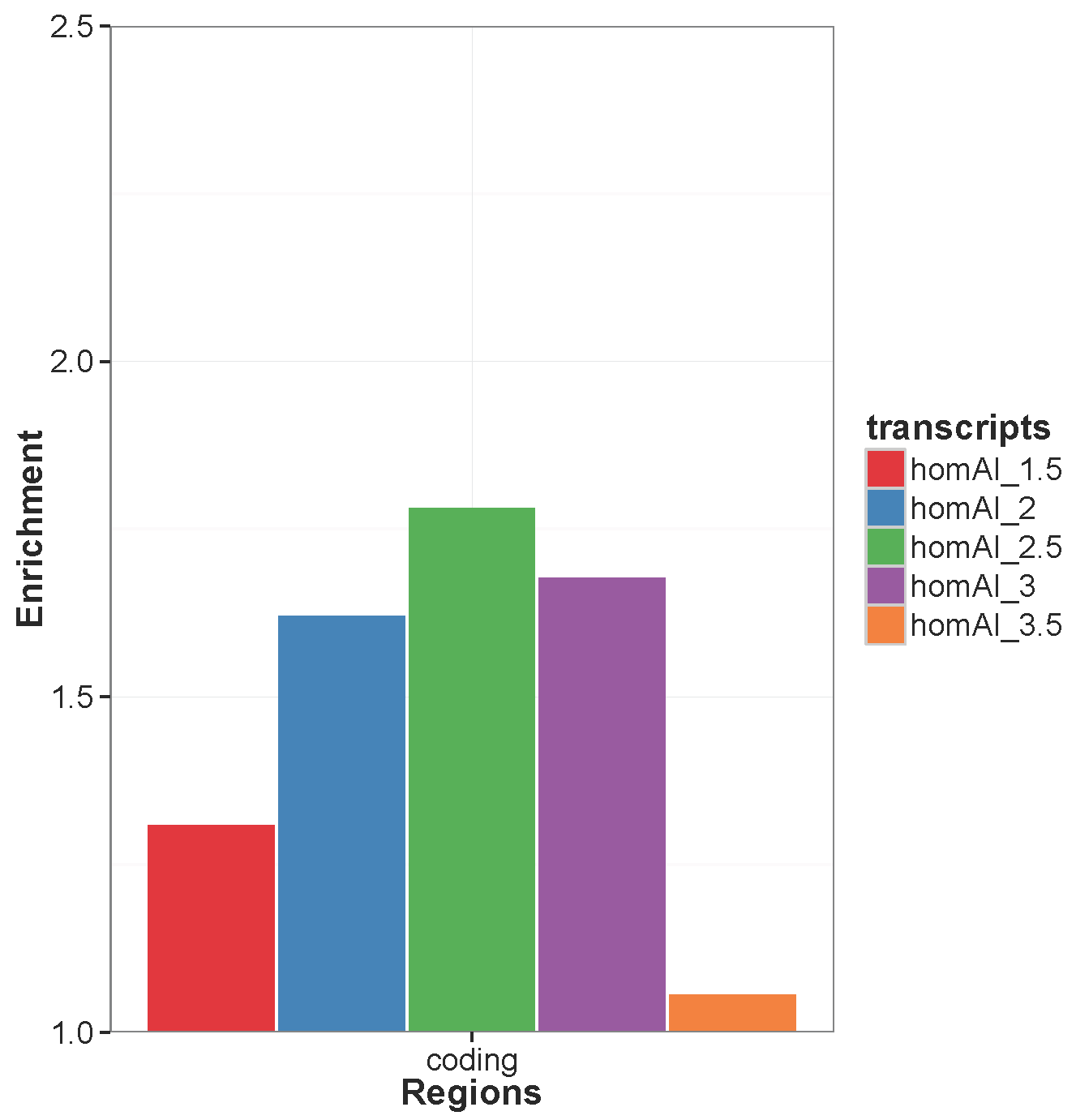


**Figure S9. Fold difference between proportions of AI transcripts with rare or novel variants in vicinity compared to AI transcripts with common variants in vicinity in the discovery set.** Only included transcripts for which the top associated SNP is homozygous (homAI). We looked at coding (histogram) vs noncoding variants around the genes (+/-20kb from gene) and in DHS regions correlated with promoters (Pearson correlation r>0.5 to 0.9). We compare different levels of allelically imbalanced transcripts from 1.5 fold to 3.5.


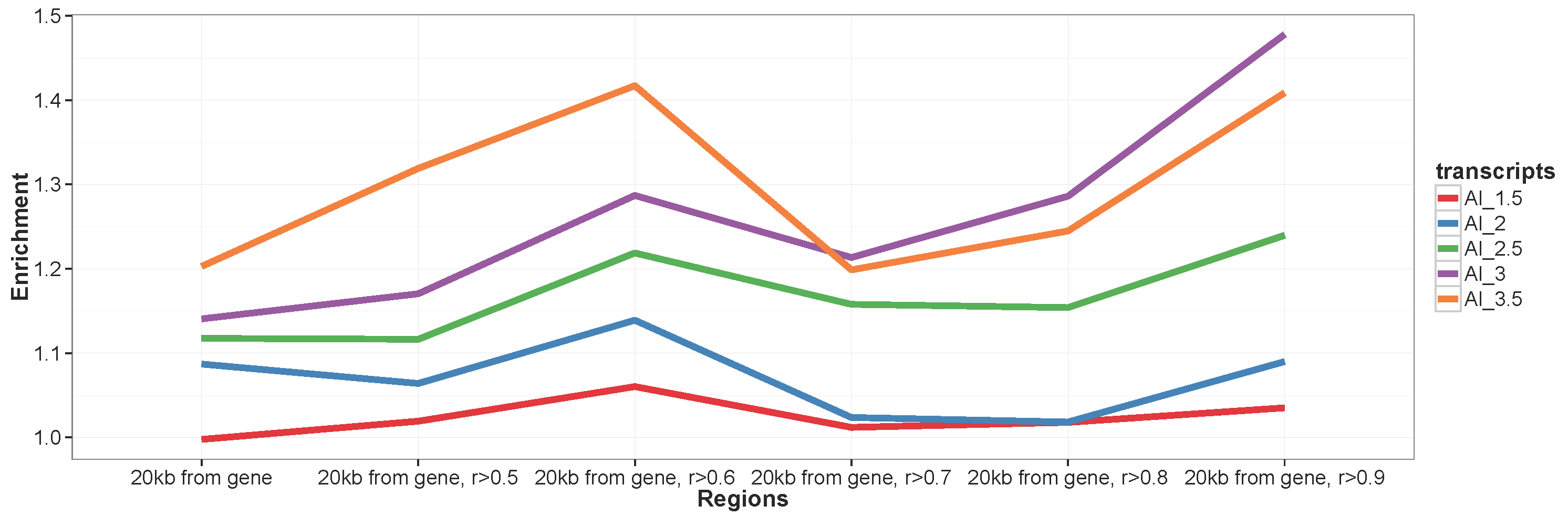

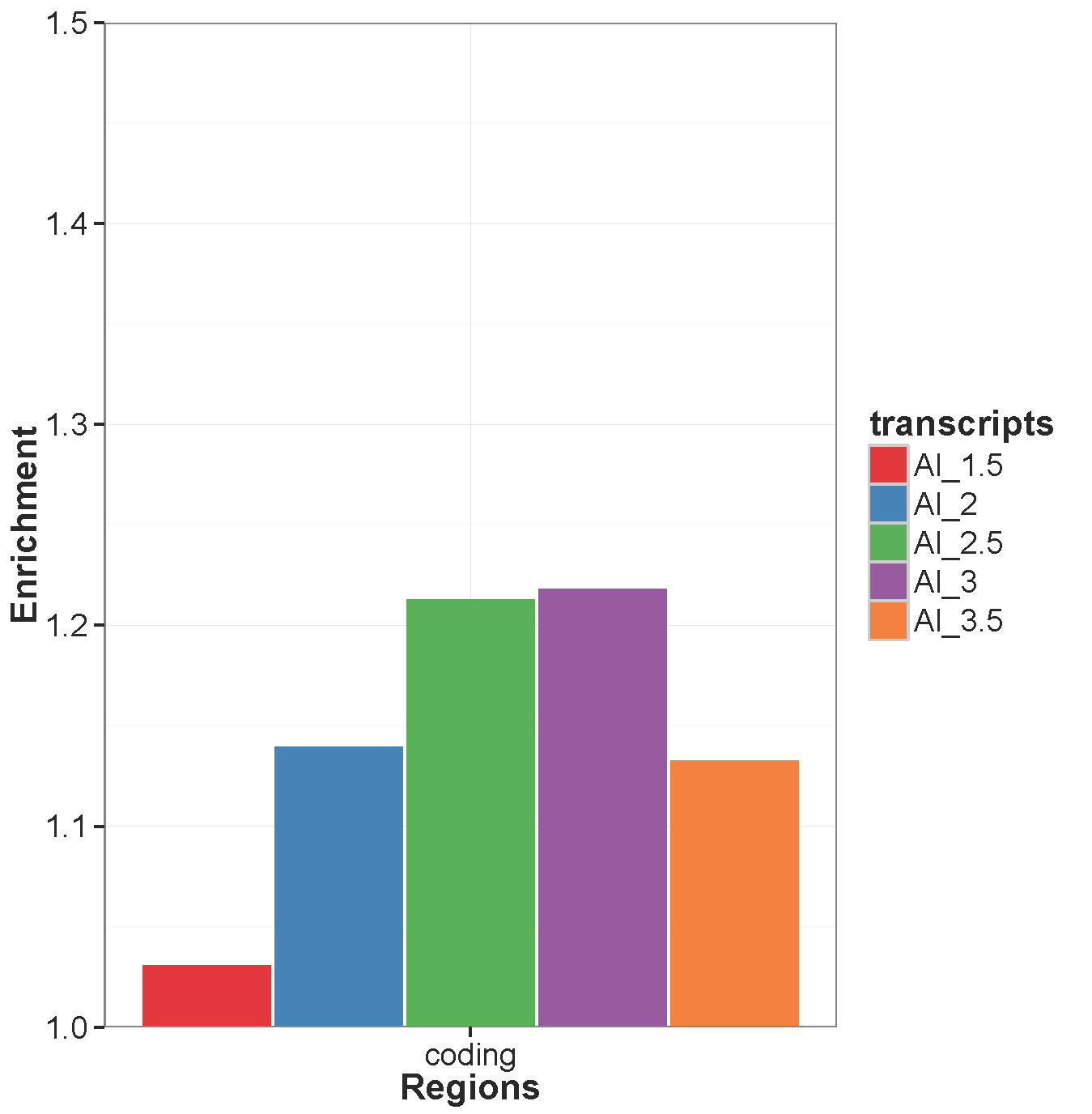


**Figure S10. Enrichment between proportions of AI transcripts with rare or novel variants in vicinity compared to AI transcripts with common variants in vicinity in the discovery set.** Including all transcripts (allAI). We looked at coding (histogram) vs noncoding variants around the genes (+/-20kb from gene) and in DHS regions correlated with promoters (Pearson correlation r>0.5 to 0.9). We compare different levels of allelically imbalanced transcripts from 1.5 to 3.5 fold.


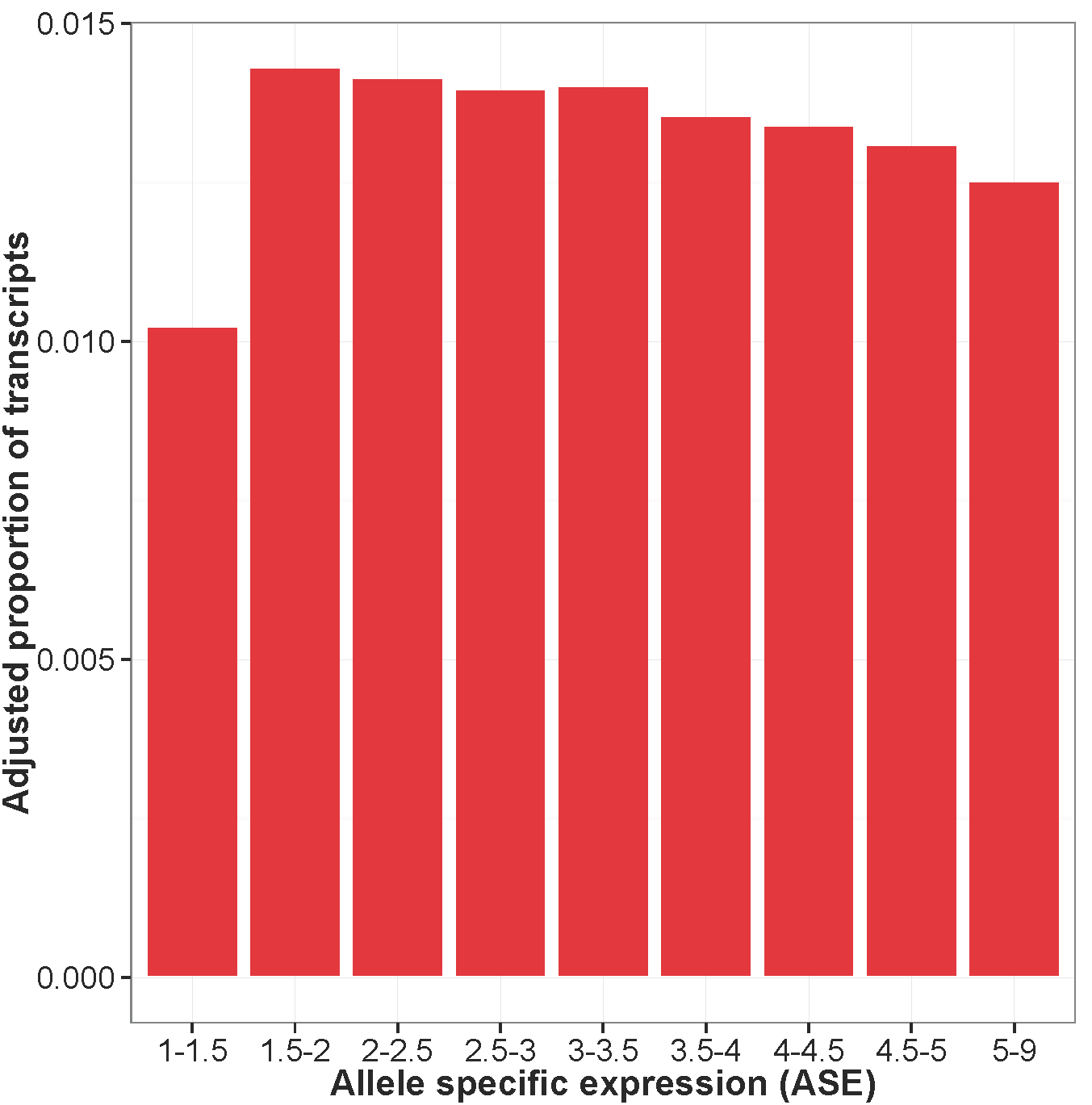

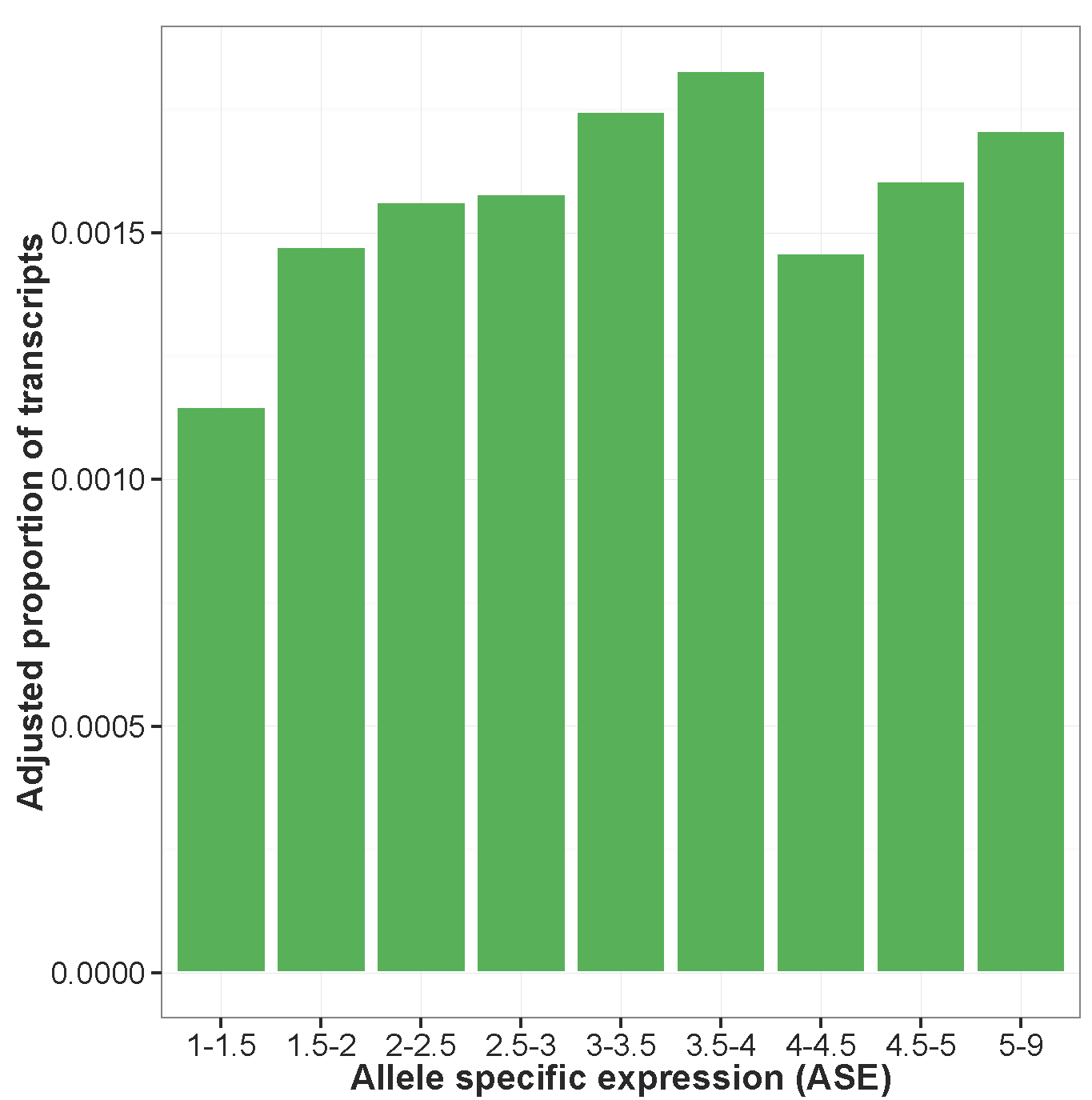

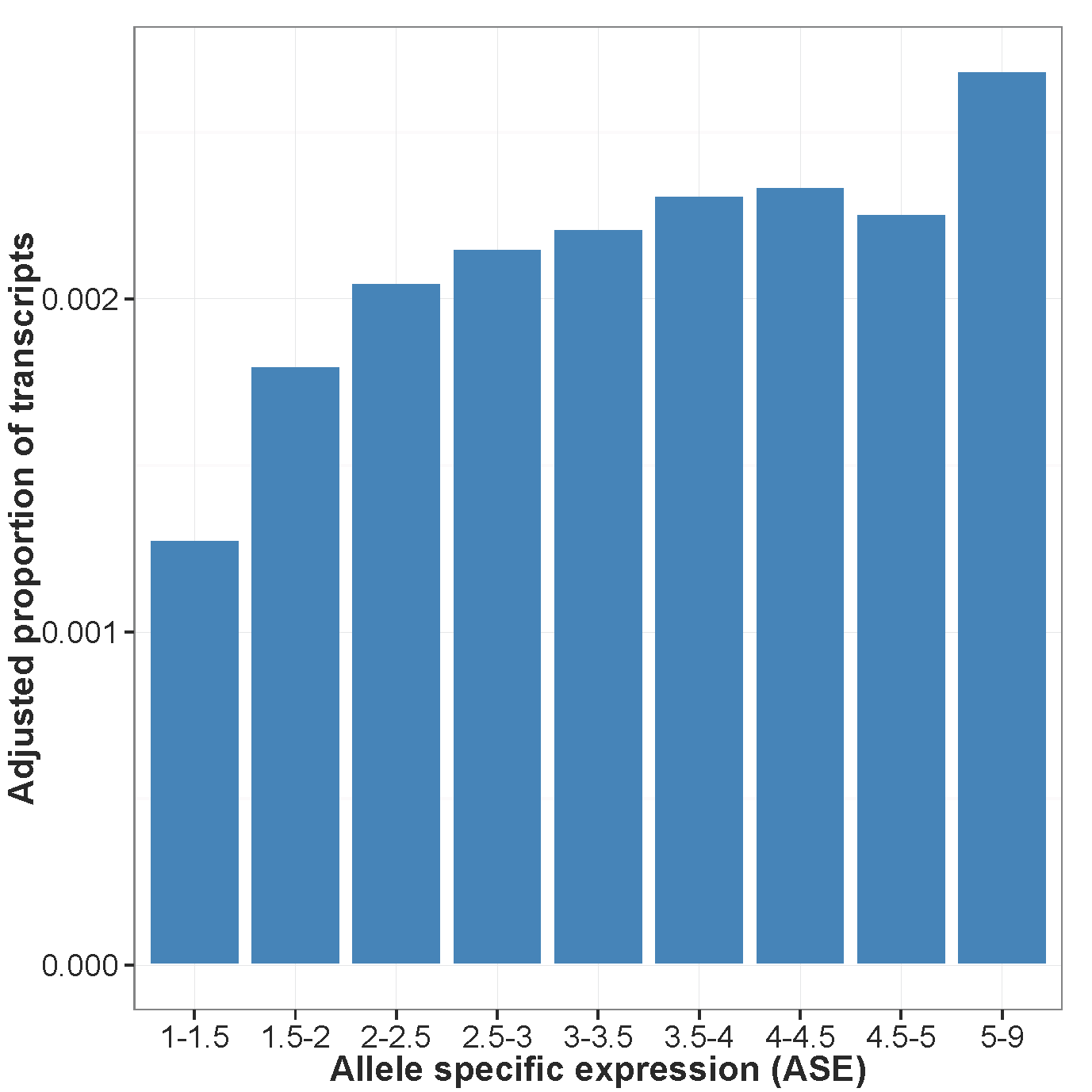


**Figure S11. Replication set distribution of allele specific expression (ASE).** Distribution of proportion of ASE in transcripts with common (red), rare (blue) or novel (green) noncoding variants in vicinity (+/-20kb from gene) adjusted for average number of SNPs used to calculate ASE

| 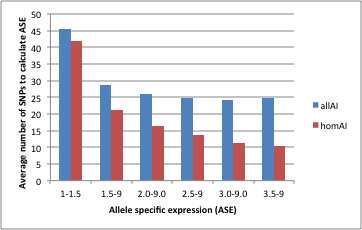 | 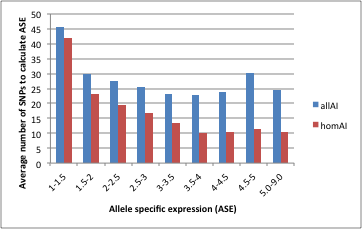 |
| --- | --- |
| **Figure S12. Average number of SNPs used to calculate allele specific expression (ASE) in the replication set**. Comparing all transcripts for which ASE was measured (allAI) and transcripts for which the top associated SNP that drives the association across samples is homozygous (homAI). | |


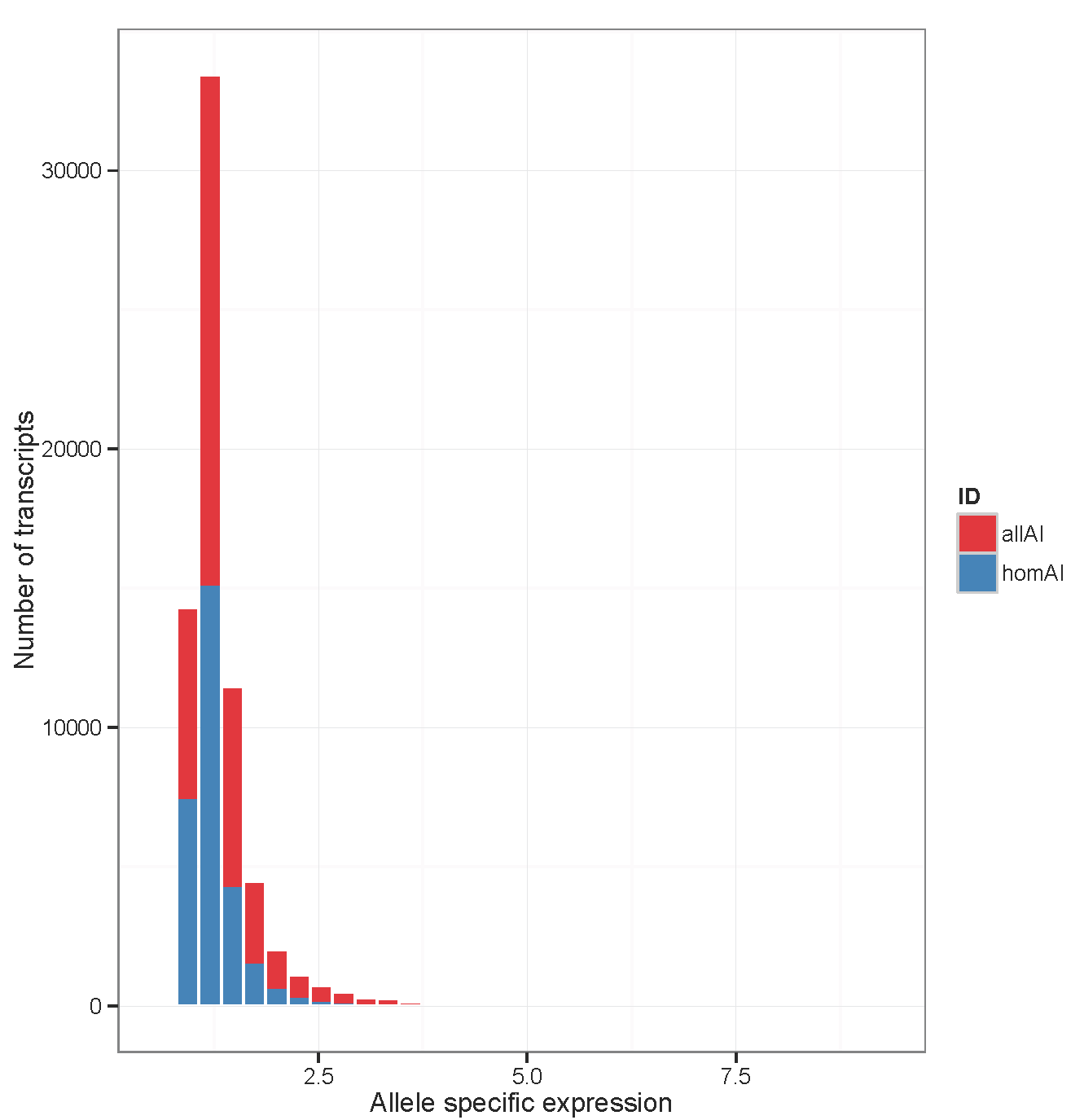


**Figure S13. Distribution of allele specific expression of all transcripts and transcripts that did not carry the common allele in a heterozygous state.** Histogram of number of transcripts from each category (overlay of the two). Comparing all transcripts for which ASE was measured (allAI) and transcripts for which the top associated SNP that drives the association across samples is homozygous (homAI).


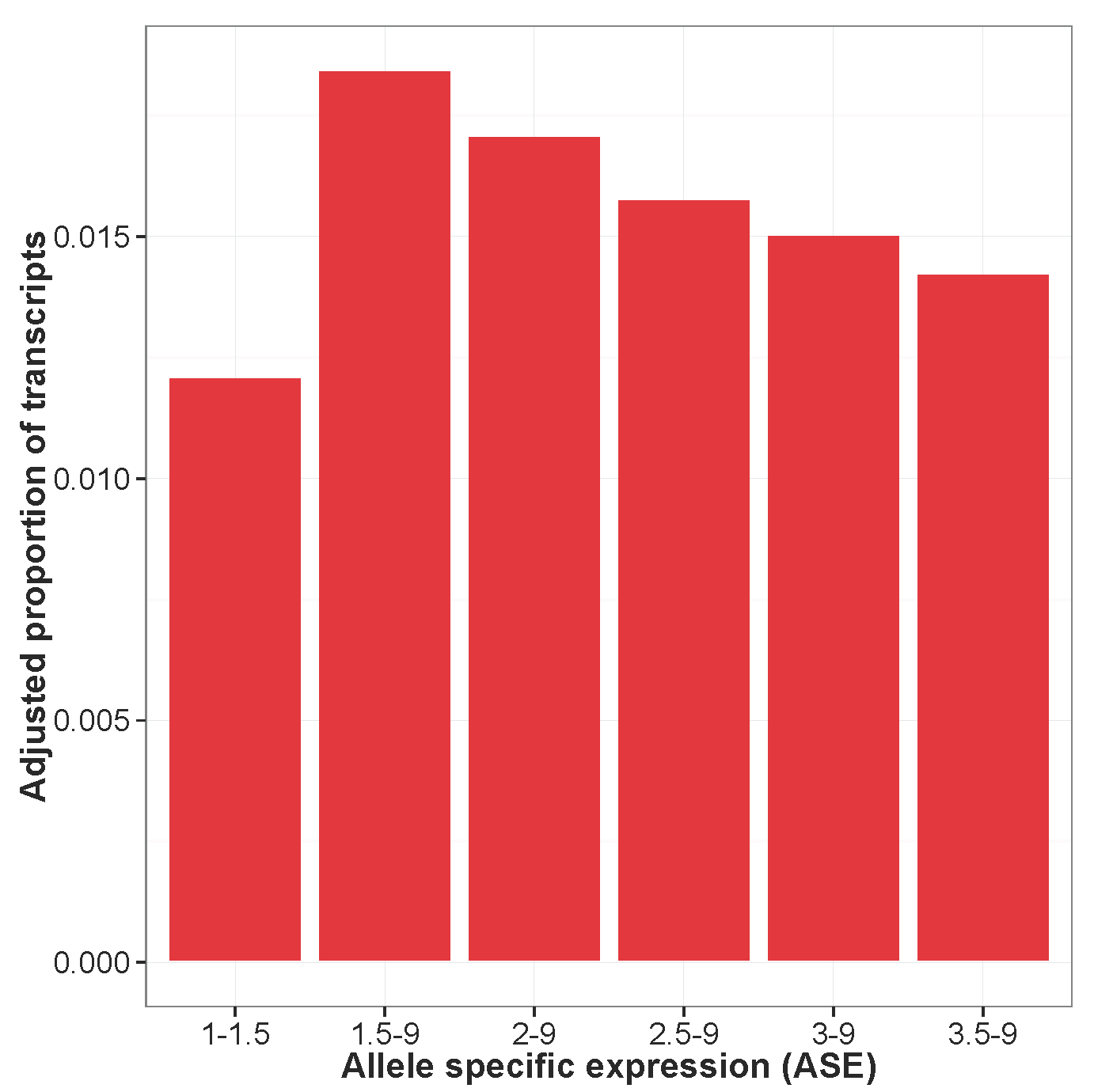

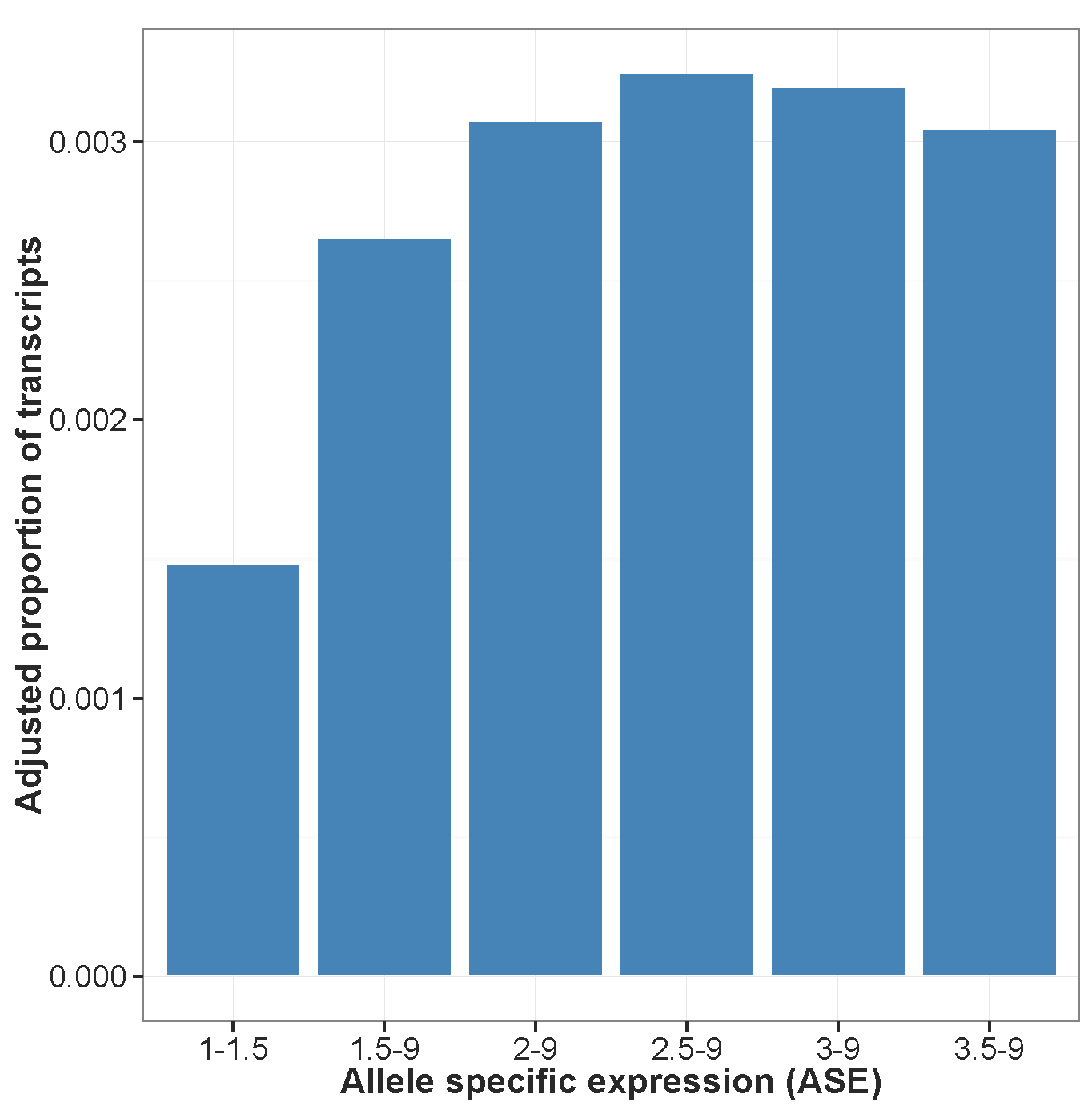

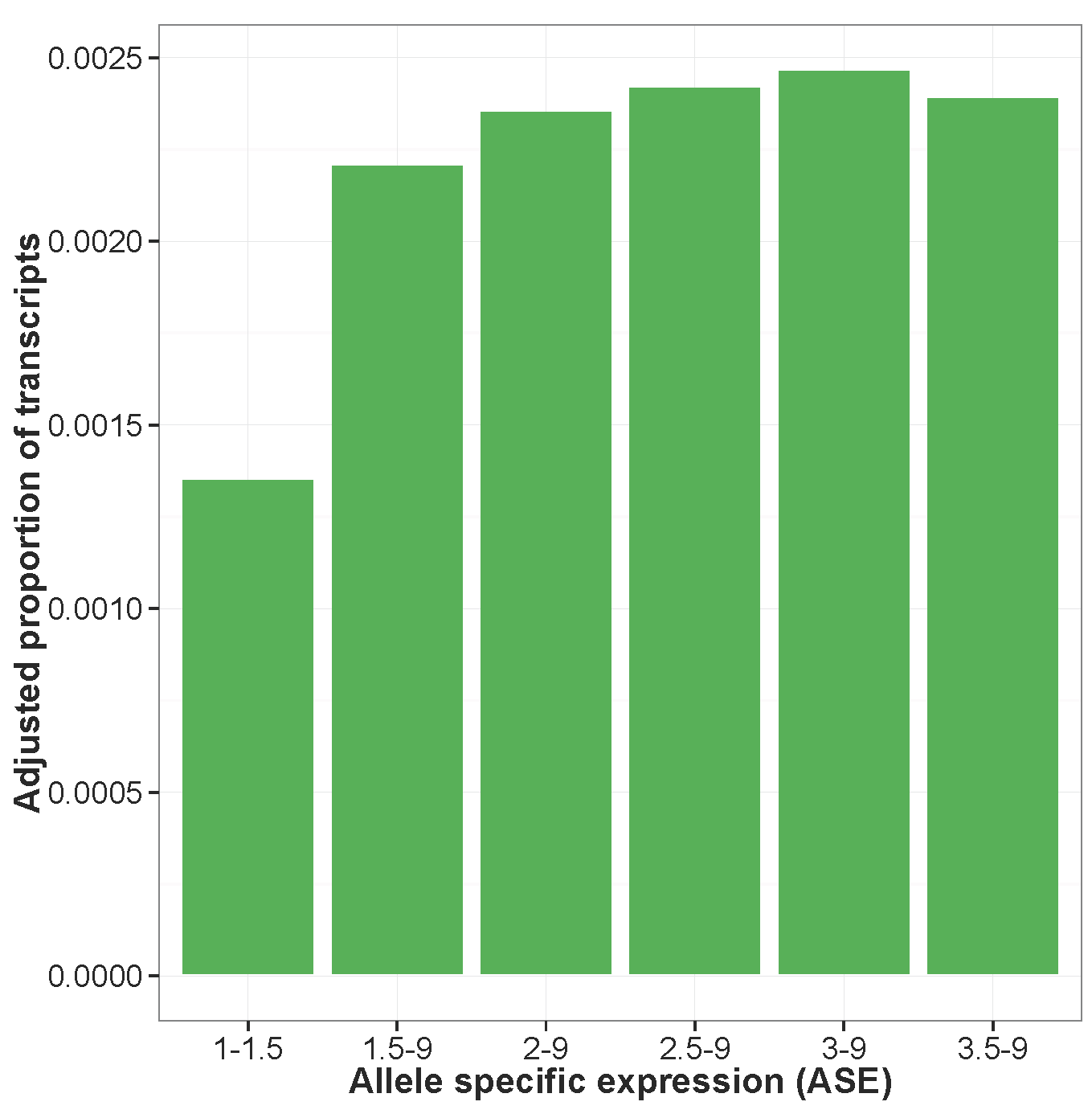


**Figure S14. Replication set distribution of Allelic Imbalance (AI).** Adjusted proportion of transcripts with common (red), rare (blue) or novel (green) noncoding variants in vicinity (+/-20kb from gene) based on different AI: 1.5 to 9, 2 to 9, 2.5 to 9, 3 to 9 and 3.5 to 9 fold difference. Only included transcripts for which the top associated SNP is homozygous in the sample (homAI).

**
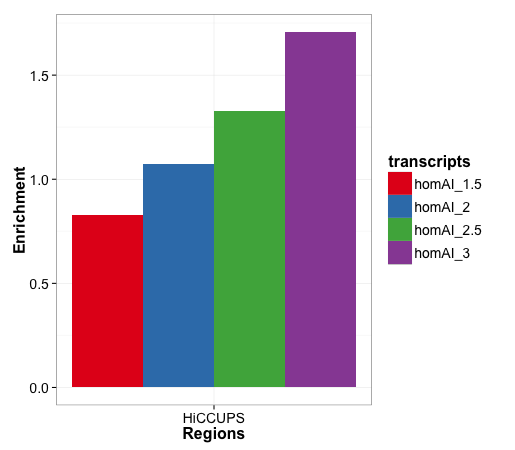
** **
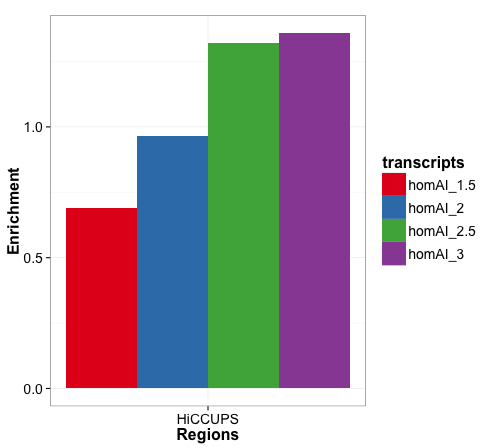
**

**Figure S15. Enrichment between proportions of AI transcripts with rare or novel variants in vicinity compared to AI transcripts with common variants in vicinity in the discovery and replication set**. Only included transcripts for which the top associated SNP is homozygous (homAI). We looked at promoter regions as well as regions linked to it by Hi-C. We compare different levels of allelically imbalanced transcripts from 1.5 fold to 3. Results from both the discovery set (left) and replication set (right) are shown.


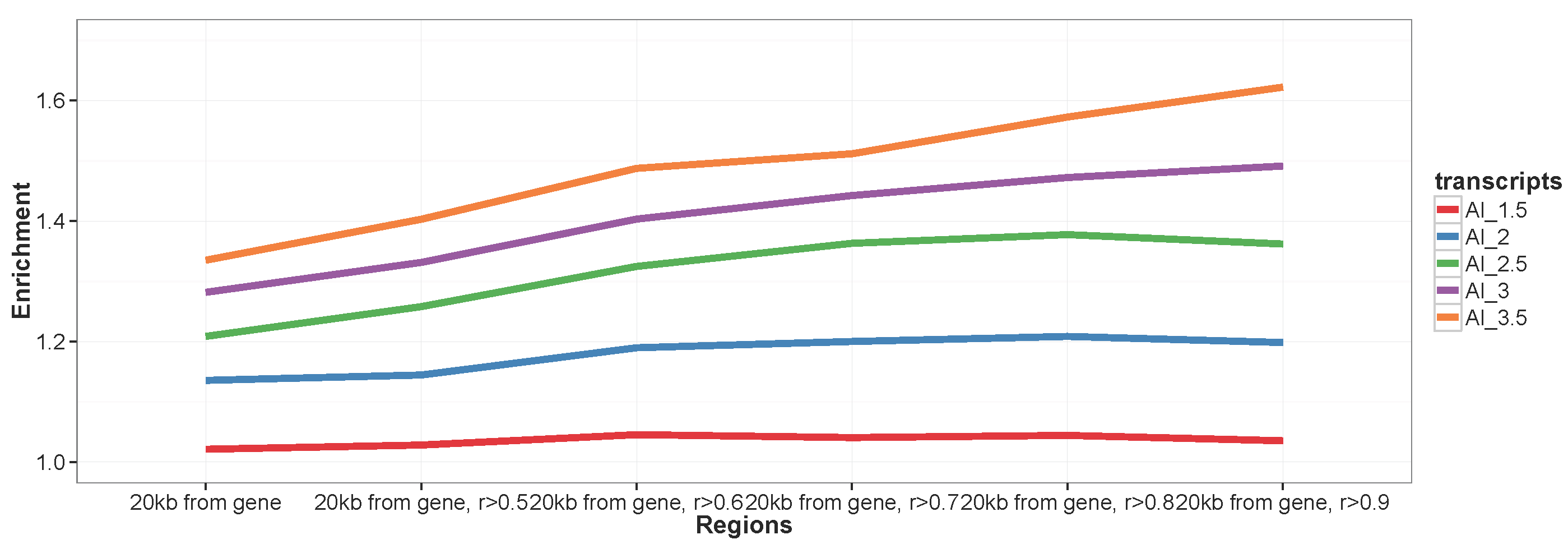

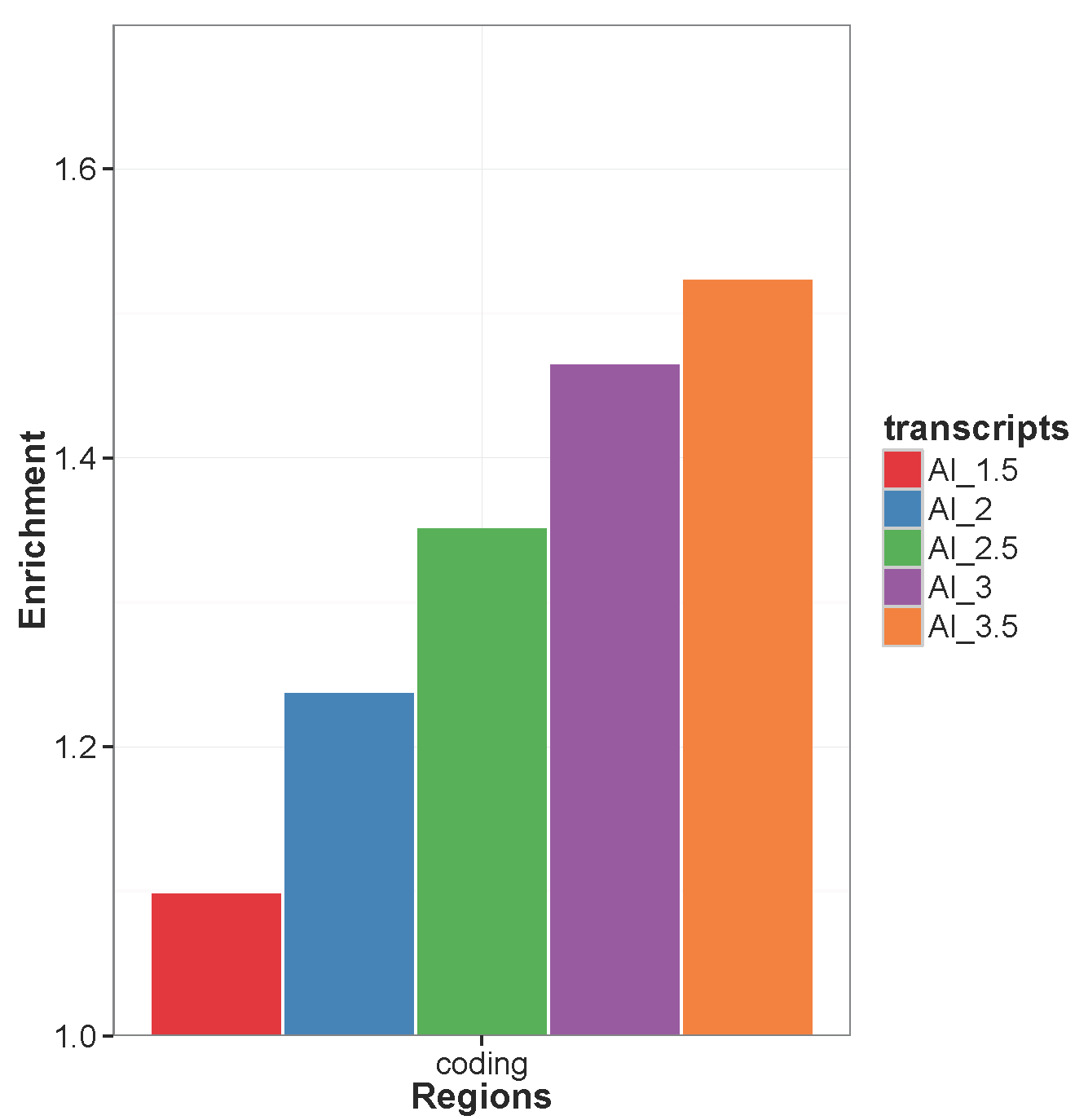


**Figure S16. Enrichment between proportions of AI transcripts with rare or novel variants in vicinity compared to AI transcripts with common variants in vicinity in the replication set**. Included all transcripts (allAI). We looked at coding (histogram) vs noncoding variants around the genes (+/-20kb from gene) and in DHS regions correlated with promoters (Pearson correlation r>0.5 to 0.9). We compare different levels of allelically imbalanced transcripts from 1.5 fold to 3.5.
